# Notch Transduction in Non-Small Cell Lung Cancer

**DOI:** 10.3390/ijms21165691

**Published:** 2020-08-08

**Authors:** Amnah Sharif, Ann Shaji, May Chammaa, Eileen Pawlik, Rodrigo Fernandez-Valdivia

**Affiliations:** 1 Department of Pathology, Wayne State University School of Medicine, Detroit, MI 48201, USA; go9818@wayne.edu (A.S.); go3089@wayne.edu (A.S.); fp3290@wayne.edu (M.C.); pawlike@med.umich.edu (E.P.); 2 Doctor of Medicine Program, Wayne State University School of Medicine, Detroit, MI 48201, USA; 3 Department of Oncology, Wayne State University School of Medicine, Detroit, MI 48201, USA; 4 Cancer Biology Graduate Program, Wayne State University School of Medicine, Detroit, MI 48201, USA; 5 Tumor Biology & Microenvironment Program, Barbara Ann Karmanos Cancer Institute, Detroit, MI 48201, USA

**Keywords:** Notch signaling, lung cancer, non-small cell lung cancer, lung development, lung cancer therapy

## Abstract

The evolutionarily-conserved Notch signaling pathway plays critical roles in cell communication, function and homeostasis equilibrium. The pathway serves as a cell-to-cell juxtaposed molecular transducer and is crucial in a number of cell processes including cell fate specification, asymmetric cell division and lateral inhibition. Notch also plays critical roles in organismal development, homeostasis, and regeneration, including somitogenesis, left-right asymmetry, neurogenesis, tissue repair, self-renewal and stemness, and its dysregulation has causative roles in a number of congenital and acquired pathologies, including cancer. In the lung, Notch activity is necessary for cell fate specification and expansion, and its aberrant activity is markedly linked to various defects in club cell formation, alveologenesis, and non-small cell lung cancer (NSCLC) development. In this review, we focus on the role this intercellular signaling device plays during lung development and on its functional relevance in proximo-distal cell fate specification, branching morphogenesis, and alveolar cell determination and maturation, then revise its involvement in NSCLC formation, progression and treatment refractoriness, particularly in the context of various mutational statuses associated with NSCLC, and, lastly, conclude by providing a succinct outlook of the therapeutic perspectives of Notch targeting in NSCLC therapy, including an overview on prospective synthetic lethality approaches.

## 1. Introduction

Notch signaling is a highly-conserved, cell-to-cell communication pathway serving several functions during mammalian lung development, including regulation of cell differentiation, survival, and lineage specification [1,2]. During early lung development, Notch promotes proximal progenitor cell types, maintaining the fundamental balance of the proximodistal axis and primarily specifying non-neuroendocrine fate choice [3,4,5,6]. Inhibition of Notch in the developing lung also produces a distal phenotype in which failure to initiate alveologenesis is observed [7,8]. In the adult lung, Notch activity continues to have a vital role with marked function in transitioning from lung developmental formation to participating in lung plasticity and repair [9]. While normal Notch signaling is necessary for maintaining homeostasis, its aberrant activity has been shown to be implicated in the onset and progression of lung carcinomas, including non-small cell lung cancer (NSCLC) [10,11,12,13], where it, furthermore, may serve of prognostic value [14,15], and as a predictor for therapy response and tumor recurrence [15,16,17]. These observations thus strongly suggest that the Notch signaling pathway may provide a promising therapeutic target in NSCLC patients. Understanding the mechanistic dynamics of Notch and the participating components hence offers insight into its involvement in NSCLC oncogenesis, as well as in its potential as a target for treatment.

**Figure 1:**
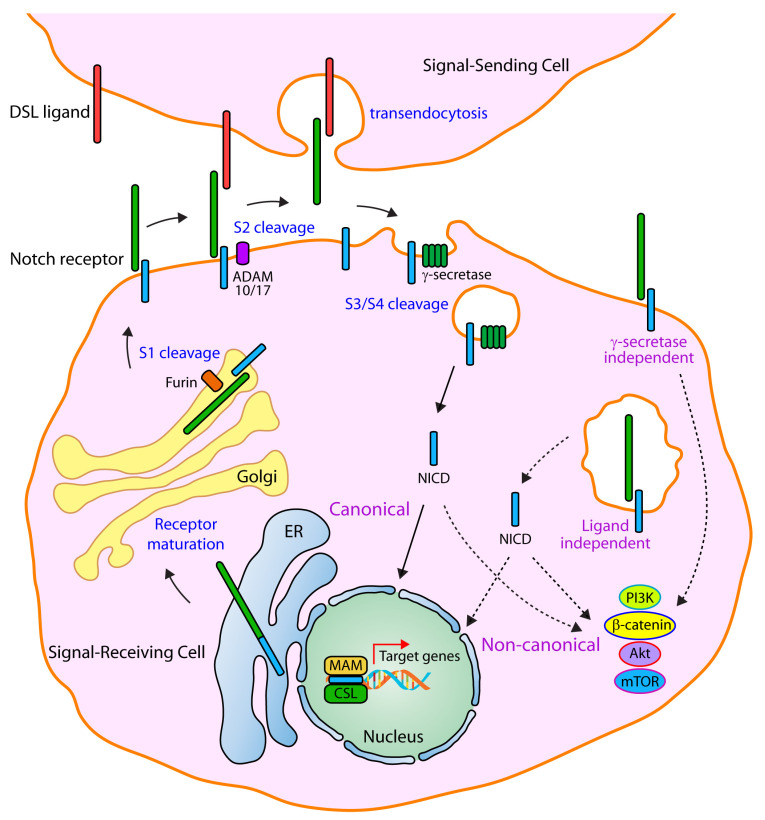
The Notch signaling pathway. Newly synthesized Notch receptors undergo post-translational maturation in the endoplasmic reticulum (ER) and Golgi apparatus. In the ER, Notch receptors are modified by several resident glycosyltransferases that add various *O*-linked and *N*-linked glycans in the EGF-repeat region of the Notch extracellular domain. Mature Notch receptors are produced in the Golgi apparatus after their first proteolytical cleavage at the S1 site, which is catalyzed by Furin-like convertases, and results in the production of heterodimeric Notch proteins. These heterodimers are thus composed of a ligand-binding Notch extracellular domain (NECD) and a single-pass Notch transmembrane and intracellular domain (NTMICD) that are tethered together through non-covalent and calcium-dependent interactions. The canonical Notch signaling cascade is initiated when Delta/Serrate/Lag-2 (DSL) family ligands present in signal-sending cells bind to the NECD of cognate Notch receptors present in apposed signal-receiving cells. Upon binding, ligand-receptor complexes initiate a transendocytosis process in the signal-sending cell, inducing a biomechanical traction that promotes a conformation change in the receptor, leading to the exposure of the S2 site, on which a proteolytical cleavage is catalyzed by the action of ADAM metalloproteases (ADAM 10/17). This ligand-dependent cleavage causes the release of a S1-S2 peptide from the NTMICD and the dissociation of the heterodimeric complex, resulting in the generation of a transient intermediate monomer in the signal-receiving cell called Notch extracellular truncation (NEXT). The S2 cleavage acts as a rate-limiting step during the initiation of the signaling cascade, and it is immediately followed by γ-secretase-mediated cleavages at the S3 and/or S4 sites. The S3/S4 intramembranous cleavage results in the release of a Notch β fragment (Nβ-peptide, sequence between S2 and S3/S4 cleavage sites) to the extracellular (or endosomal) space, and the Notch intracellular domain (NICD) into the cytoplasm of the signal-receiving cell. The released NICD then initiates a translocation journey to the nucleus, where it participates, along with the DNA-binding protein CSL (CBF1/RBPjκ/Su(H)/Lag-1), Mastermind (MAM)-like proteins and other co-activators, in the formation of transcriptional complexes that initiate the expression of Notch downstream effectors of the Hes and Hey transcription repressor families. In its most generic form, non-canonical Notch signaling (dotted arrows) is independent of CSL and, instead, mediated through interaction with other signaling axes including PI3K, mTOR, Wnt and Akt; and could be triggered through either ligand-dependent or ligand-independent mechanisms, and could also occur in γ-secretase-dependent as well as in γ-secretase-independent modes. Notably, ligand-independent activation of Notch receptors, which mainly occurs in endosomal trafficking routes and multivesicular bodies (MVB), results in the production of NICD, which in-turn could prompt either CSL-dependent as well as CSL-independent signaling responses.

Mammals express four Notch receptors (Notch1 to Notch4) and five ligands: Delta-like 1,3,4 (Dll1, Dll3, Dll4) and Jagged 1,2 (Jag1, Jag2) [18,19]. Notch receptors are comprised of three main domains: Notch extracellular domain (NECD), Notch transmembrane domain (NTM) and Notch intracellular domain (NICD). Notch signaling is activated when a transmembrane ligand on a signal-sending cell interacts with the NECD of a transmembrane Notch receptor on a signal-receiving cell, inducing a conformational change in the receptor (Figure 1). Endocytosis of the ligand in the signal-sending cell then allows the deployment of a quantum catch-bond pulling force between the signal-sending cell ligand and the bound signal-receiving cell receptor [19,20] that results in exposing the S2 site of the Notch receptor to an ADAM (a disintegrin and metalloprotease) protease (ADAM 10/17), which cleaves the extracellular domain of the Notch receptor and allows both endocytosis of the NECD-ligand complex in the signal-sending cell (NECD transendocytosis) and the initiation of a subsequent proteolytical activation process in the signal-receiving cell [18,19]. The S2 cleavage is followed by an intramembranous proteolytic cleavage (either at the cell membrane or endosomal vesicles) catalyzed by the gamma secretase complex at the S3/S4 site, releasing both the NICD intracellularly and the Nβ peptide (equivalent to amyloid β42) to either the interstitial space or the endosomal vesicle lumen. Upon its proteolytical release, NICD translocates to the cell nucleus, where it interacts with CSL (Rbpjk), Mastermind-like proteins, and p300, engaging in a coactivator complex to transcriptionally activate basic helix-loop-helix (bHLH) genes, such as the *Hes* and *Hey* family members of transcriptional regulators, which then exert their biological effects (Figure 1).

In this review essay, we first start by providing a deep mechanistic overview of Notch function in lung development and its driving role in the dynamics of lung cell populations’ ontogeny, fate decisions, and differentiation processes, as this fundamental mechanics seems now evident that is well conserved in the molecular and cellular dynamics triggering lung cancer initiation and neoplastic progression, then provide a conceptualized revision of Notch’s involvement in NSCLC formation and progression, particularly in attention to the tumor promoter and/or suppressive roles of distinct Notch receptors, ligands and key regulators, and within the contexts of diverse cell ontogeny and fate choice selection, dynamic interplay interactions with other pathways, mutational status, genetic and epigenetic regulation, and on their potential as actionable drivers in disease progression and as targets for therapy, and, finally, include a concise discussion on prospective Notch targeting therapies, subclinical and clinical testing studies, and on how these strategies could be integrated for synthetic lethality approaches and other combinational modalities.

## 2. Notch Signaling in Lung Development

The lung is a highly complex structure of branching airways responsible for facilitating gas exchange. The lung develops across three general time periods, embryonic, fetal, and post-natal, with the developmental process categorized morphologically into five stages, as follows: embryonic, pseudoglandular, canalicular, saccular, and alveolarization [21]. The two driving processes that foster lung development through these stages are branching morphogenesis (structural development of the airways) and alveolarization (functional development of the respiratory epithelium via alveoli formation to facilitate gas exchange) [21].

The embryonic stage of lung development begins with lung cell fate determination as the foregut endoderm and mesoderm germ layer give rise to the lung epithelium and surrounding mesenchyme, respectively [22]. The lung mesenchyme promotes sprouting and branching and interacts with the endoderm to manifest various cell lineages including connective tissue, endothelial cell precursors, and smooth muscle [23]. At approximately four weeks post-conception in humans [24], embryonic day 9.5 in mice (E9.5), evagination of Nkx2.1^+^ epithelial cells into the surrounding mesenchyme stems the formation of the right and left lung buds, which subsequently undergo rapid differentiation to form epithelium-lined airways following a highly regulated process known as branching morphogenesis [22,23]. The pseudoglandular phase of lung development (E12.5-E16.5) continues the expansion of these airways from the proximal region (trachea and bronchi) to form distal airway structures (bronchioles), with domains of proximal and distal progenitors marked by the mutually exclusive expression of Sox2 and Sox9/Id2 proteins, respectively [23], and the lateral inhibition-mediated specification of neuroendocrine and non-neuroendocrine fates [3,8]. The culmination of the bronchiole proliferation is visibly marked by the canalicular phase (E16.5-E17.5), where the terminal bronchioli begin to differentiate into the acinus, a cluster of epithelial cells (initially formed during the pseudoglandular stage) that will eventually become the functional unit for the alveolus [21,23]. Also, as hallmarks of the canalicular phase are the club vs. ciliated cell selection and the evolution of distal epithelial cells into alveolar type 1 (AT1) and type 2 (AT2) cells from a common bipotent progenitor [25,26,27]—although, it should be noted that it is also well established that AT2 cells can act as progenitors for AT1 cell differentiation in the postnatal lung [22,23,28]. AT2 cells are smaller, cuboidal cells primarily responsible for the production and secretion of pulmonary surfactant onto the alveolar surface which noticeably allows the alveolus to exist in homeostasis [29], while AT1 cells are large squamous cells that make up 95% of the alveolar surface and mainly function in gas exchange [29,30]. Further in the organogenesis, the saccular stage (E18.5 to postnatal day 5 (P5)) accounts for a transitional period during which the acini widens and forms saccules (clusters of airspaces separated by primary septa), in a concerted process involving T1α, Hikeshi (l7Rn6), Nfib, and Fox proteins Foxa2 (HNF-3β) and Foxm1 [21,23,31,32,33,34,35]. Notably, unlike humans, mice and other insessorial mammals are born during this stage of lung development, with alveologenesis occurring postnatally [21,24]. At the alveolarization stage, the saccules are further divided by secondary septa into smaller airspaces called alveoli, in an orchestrated interplay between myofibroblasts, endothelium and alveolar epithelial cells that is finely regulated by PDGF-A, FGFR3/4 and VEGF interactions [36,37,38], and greatly increases the surface area for gas exchange.

*Notch1* transcripts are initially present in the proliferating tips of budding lung epithelium and surrounding mesenchyme at E10, marking, along with the expression of *Hes1* and *Hey2* mRNAs in the budding epithelium, the first detectable levels of Notch activity in the developing lung [5,39]. Interestingly though, it has also been observed that by E12 mRNAs for all *Notch* receptors, both *Jagged* ligands, and *Dll4* (which, in the epithelium, is present in the proximal but not distal compartment at this stage) were expressed in the lung mesenchyme as well [5]. The dynamic activity of Notch signaling observed throughout the developing lung strongly suggested that the Notch pathway could indeed be playing a pivotal role in lung morphogenesis and initial studies through the use of mouse platforms deficient for Notch pathway components and pharmacological inhibition of Notch activity were thus carried out [3,5,6,40]. Through the use of DAPT, a gamma secretase inhibitor (GSI) that prevents gamma secretase-mediated cleavage of Notch receptors [41], Tsao at el. demonstrated that the blockade of proteolytical Notch processing in murine lung primordium explants resulted in lung outgrows that exhibited significantly reduced levels of *Sox2* [5], a factor present in proximal progenitors that are necessary for the generation and maintenance of several cell lineages, including basal and club cells [42]. In these studies, it was markedly shown that while lung buds were formed by E8.5 and the distal region appeared substantially enlarged, impaired growth in the proximal region was observed [5]. Interestingly, an additional phenotypic manifestation noted in these DAPT-treated lung explants was ectopic budding in the proximal region and significant up-regulation of *Fgf10* in the surrounding mesenchyme, which, along with the observed inductive effect of *Fgf10* on epithelial Notch activity, further indicated not only a suppressive effect of Notch signaling in *Fgf10* expression, but also the existence of a Fgf10/Notch counterbalance dynamic regulatory loop in early lung morphogenesis [5].

**Figure 2:**
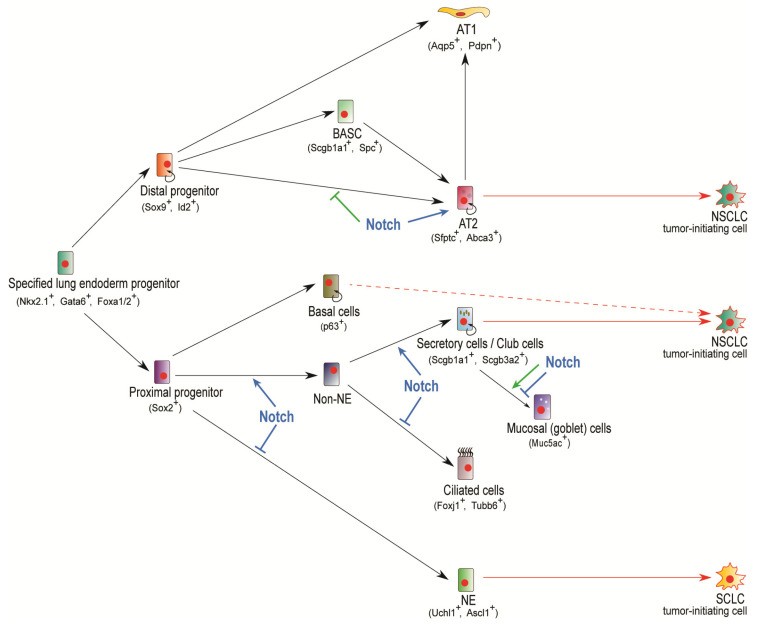
Notch signaling in lung development and malignant conversion. Schematics depicting Notch-mediated cell specification and fate choice in lung development. In proximal progenitors, Notch regulates neuroendocrine (NE) vs. non-neuroendocrine (Non-NE) fate choice by inhibiting neuroendocrine commitment while promoting Non-NE fate. Once established, Non-NE progenitors are also under Notch-controlled fate determination, where Notch activity promotes secretory fates while suppressing ciliated cell fate choice. Goblet cells, which can derive from secretory/club cells, are increased in numbers upon augmented Notch activity (Notch1^ICD^ overexpression, green arrow). Surprisingly, and contrastingly, a Notch suppressive action is revealed from studies of loss of either Pofut1, Rbpjk or Jag1, where Goblet metaplasia is manifested. Of note, Notch2 and Jag1 blocking antibodies are known to halt and reverse ovoalbumin- and cytokine-induced Goblet cell metaplasia. AT2 trajectory is negatively controlled by enhanced Notch signaling (green line-bar inhibition symbol) as revealed by the terminal cell differentiation defect observed upon Notch3^ICD^ and Notch1^ICD^ overexpression. Interestingly however, Notch activity is required for AT2 cell maturation and survival once formed. Neuroendocrine cells give rise to small cell lung cancer (SCLC) whereas both AT2 and club cells have been shown to act as cells-of-origin for NSCLC (both ACL and SCC) and basal cells are also presumed to give rise to SCC.

Pofut1 (protein *O*-fucosyltransferase 1) is responsible for *O*-fucosylating Notch receptors at their extracellular domain, one of the numerous processes required to produce a viable Notch receptor able to successfully bind to ligands and activate Notch signaling [43]. While *Shh-Cre*-directed conditional knockout of *Pofut1* in murine lung yields a similar phenotype as with GSI treatment, *Pofut1^cNull^* (conditional knockout) lungs also show deficiency of club cell secretory lineage accompanied by an overpopulation of ciliated cells and neuroendocrine cells [6]. Importantly, these interesting findings, along with the observed neuroendocrine cell pool expansion and club cell reduction displayed in *Hes1* deficient mice [3], and the fact that Sox2 was significantly downregulated in E18.5 *Pofut1^cNull^* lungs [6], strongly suggest that Notch selectively suppresses ciliated and neuroendocrine cell identities and likely controls club cell populations by promoting the expression of cell-autonomous, proximal progenitor gene, *Sox2*. Indeed, Sox2 has been shown to be essential for club cell differentiation and proximal cell types genesis [44]. A necessary remark in these observations however, is the fact that at E14.5, Sox2 labeling of *Pofut1^cnull^* lungs was essentially comparable to control lungs in both the pattern and number of labeled cells [6]. Interestingly, the studies by Tsao et al. also demonstrated that *Jag1* was expressed in ciliated cells in a salt and pepper pattern, and that this local expression configuration was abolished upon Notch abrogation in *Rbpjk^cNull^* (*Shh-Cre;Rbpjk^flox/Δ^*) mutant mice where virtually all epithelial cells became *Jag1* positive [6]. This information, presumably indicating a lateral inhibition mode of action, was further confirmed by the studies of Morimoto et al., where both a mutually exclusive distribution of Notch1^ICD^ and Foxj1 in the epithelial compartment, and an altered fate specification in which practically all Rbpjk-deficient proximal cells scored positive for the ciliated cell marker Foxj1 were seemingly demonstrated [40]. Of crucial importance, the lineage tracing experiments conducted by Morimoto et al. through combination of Notch activity reporter *N1IP::CRE* and conditional *R26R* Cre activity reporter, allowed conclusive demonstration that Notch signaling action is indeed necessary for club cell ontogenesis [40], in a process that, furthermore, requires Jag1-mediated activation of Notch receptors [45]. Collectively, these observations indicate that Notch signaling is required for non-neuroendocrine fate specification and the genesis and selection of club cells. In the absence of Notch activity, fate determination is pulled towards a default neuroendocrine fate specification, and during club cell ontogenesis towards a ciliated cell program (Figure 2).

*SPC-Cre;Notch1^ICD^* transgenic mouse embryos, which overexpress Notch1^ICD^ throughout the lung epithelium under surfactant protein C (SPC) promoter governance, develop dilated cysts in place of normal saccules, and high levels of Hes1 are markedly observed not only in the proximal epithelium but also passed through the bronchioalveolar duct junction (BADJ) [4]. In this model, alveolar differentiation of epithelial cells is severely affected and areas of aberrant Nkx2.1^+^Ecadherin^+^HNF-3β^+^ epithelial cells are seen in the distal epithelium [4], an effect that is in line to the AT1 and AT2 differentiation defect observed upon directed overexpression of Notch3^ICD^ in the pulmonary epithelium [46], where, furthermore, an accumulation of abnormal, TTF-1^+^ cuboidal undifferentiated cells is observed [46]. As opposed to Notch1 inactivation, which renders the lung devoid of club cells, the *SPC-Cre;Notch1^ICD^* overexpressing transgenic mice also displayed ectopic presence of CC10^+^ club cells in the distal cystic regions of their lungs along with marked metaplasia of mucous cells and decreased numbers of ciliated cells [4], further reaffirming not only the club fate promoting role of Notch activity, but also revealing a putative plausible role in promoting mucous cell differentiation among the non-neuroendocrine lineages/progenitors. Contrastingly however, recent findings on models with conditional inactivation of *Rbpjk* or *Pofut1* via *Tgfβ3-Cre* show that in absence of canonical Notch activity, Goblet cell metaplasia is induced in the postnatal lung [47]. Although intuitively difficult to conceal the above observations, it’s been proposed that different thresholds of Notch activity at distinct topological and developmental frames may account for the converse action of Notch activity in Goblet cell formation [47]. Intriguingly, and to further complicate this picture even more, Notch2 has been shown to be required for cytokine-driven Goblet cell metaplasia in adult lung [48], while studies with *Jag1* inducible conditional deletion (*SPC-rtTA;Tet-O-Cre;Jag1^flox/flox^*) and Jag1 blocking antibodies gave converse results indicating, respectively, that Jag1 deficiency induces mucous cell metaplasia at the expense of club cells [45], and that antibody-mediated Jag1 blocking is able to reverse ovoalbumin-induced, Goblet cell metaplasia [49]. Although the above-mentioned observations indicate that Notch-regulated Goblet cell formation seems to be a more intricate process, they are, nonetheless, consistent with the ascribed major role of Jag1-Notch2 action in club cell function and fate [50]. Notwithstanding, what however is clear now from lineage tracing analysis in adult lungs with the *Tgfβ3-Cre/+;Pofut1^flox/flox^;G-Red/+* system [47] and transdifferentiation analysis in *Hoxa5* deficient mice [51] is that Goblet cells can originate from a distinct subpopulation of club cells in the proximal epithelium.

One of the most fascinating discoveries in the lung morphogenetic process was the recent unveiling of cell slithering in the ontogenesis of pulmonary neuroendocrine cells and bodies (NEB) [52,53]. In elegant studies by the Krasnow and Morimoto laboratories, it was demonstrated that neuroendocrine epithelial cells adopt displacement characteristics of EMT (epithelial mesenchymal transition) to migrate towards one another and coalesce as clusters within the developing epithelium [52,53]. While for long considered to be derived from the neural crest, it is now accepted that this epithelial lineage originates from tip endoderm progenitors, and is remarkable for displaying high levels of the proneural bHLH transcription factor Ascl1 (Mash1) and the Notch ligand Dll1 in a local topographic setting where its clusters are surrounded by SPNC (SSEA1^+^, peri-NEB, Notch-active, CC10^−^ [50]) cells [53]. Remarkably, 3D mapping of neuroepithelial bodies (NEB) in developing lungs conditionally deficient for *Hes1* (*Shh-Cre;R26R^H2B-mCherry^;Ret^EGFP^;Hes1^flox/flox^*) demonstrated enlarged NEBs throughout the proximal to distal airways, indicating that Notch-Hes1 signaling is required for restricting neuroendocrine cell fate in epithelial progenitors [53]. Strikingly, when SPNC cells are selectively eliminated through inducible diphtheria toxin expression (*Nkx2.1-Cre^ERT2^;UPK3-STOP-DTA*) at E14.5, the number of neuroendocrine cells remained unchanged [53], indicating that these (SPNC) Notch-active cells do not contribute to the neuroendocrine cell fate when neuroendocrine cells have formed, and, hence, strongly suggesting that the Notch/Hes1 restriction occurs through a lateral inhibition mechanism, a process that is also further in line with previous observations indicating that neuroendocrine cells are markedly positive for the Notch ligand Dll1, and that concomitant genetic inactivation of Notch1, 2 and 3 (*Shh-Cre;Notch1^flox/flox^;Notch2^flox/flox^;Notch3^−/−^*) results in SPNC absence and subsequent NEB expansion [50]. Of interest, a recent report on Notch ligands’ dynamics function has shown that while Jagged ligands had significant roles in the control of cell populations present in conducting airways, it is Delta-like ligands the ones that are relevant on the control of NEB formation and size [54]. Using *Shh-Cre;Jag1^flox/flox^;Jag2^flox/flox^* mice, the Cardoso group was able to demonstrate that the deficiency of Jagged ligands—which furthermore were also shown in this study to be expressed in a proximo-distal wave pattern in the lung epithelium—did not result in marked alterations in NEBs’ distribution, Cgrp expression, or cluster extent size in the intrapulmonary airways, while *Shh-Cre;Dll1^flox/flox^;Dll4^flox/flox^* animals displayed marked enlargement of Ascl1^+^ neuroendocrine cell pool, which, moreover, was further modestly seen in animals conditionally deficient for *Dll1* but not in ones for *Dll4*, thus markedly suggesting that while *Dll1* is the main Notch ligand responsible for suppression of neuroendocrine fate in the developing epithelium, functional redundancy between these ligands exists in the control of neuroendocrine cell fate specification [54]. These observations are however in marked contrast with Keli Xu group’s findings indicating that Jag1 inactivation results in an increase in the number of Cgrp^+^ cells in the distal lung [45]. Importantly, while the distinct strategies used by Zhang et al. and Stupnikov et al. to genetically inactivate *Jag1* in the developing lung epithelium may account for these divergent results, conditional genetic ablation of global Notch regulators *Pofut1* and *Rbpjk* results in a noticeable distinct degree of NEB expansion [6,40], and it’s been proposed that Hes1 expression—which is known to control NEB fate [3] and yet is maintained in Rbpjk null epithelium [40]—might be regulated through Notch-independent mechanisms. While this is a valid idea, the notion for non-canonical and/or ligand-independent Notch action has not been ruled out either.

Lunatic fringe (*O*-fucosylpeptide 3-beta-N-acetylglucosaminyltransferase, Lfng), a key modifier in the Notch signaling pathway responsible for transferring N-acetylglucosamine glycans to *O*-linked fucose residues present in epidermal growth factor-like (EGF) repeats of Notch receptors [55], regulates Notch activity by simultaneously making Notch receptors more sensitive to binding with Delta-like ligands and less sensitive to Jagged ligands [56,57]. *Lfng* knockout mutant mice manifest dramatic defects in lung vasculature and branching, including delayed sacculation, deformed septation and aberrant alveolar development [8]. Notably, an increase in AT2 cells fraction (assessed by SPC expression), a decrease in AT1 markers, and absence of SMA^+^ myofibroblast cells were observed in these mutants at E17.5 developmental stage [8]. Critically, the latter manifestation, was shared by *Notch2^+/−^;Notch3^−/−^* compound mutants, but not displayed neither in *Notch2^+/−^* nor in *Notch3^−/−^* monogenic animals [8], clearly indicating that Notch2 and 3 work redundantly in myofibroblast differentiation. Interestingly, recent studies by the Kopan group on Notch1^ICD^ and Notch2^ICD^ equivalency have demonstrated that Notch actions are mainly dependent on NICD dosage rather than on NICD type [58]. Whereas inducible conditional deletion of *Rbpjk* (*ROSA26-rtTA;Tet-O-Cre;Rbpjk^flox/flox^*) in embryos at E14.5-E18.5 resulted in myofibroblast defects without affecting alveolar epithelial cells [8], thus plausibly implying a primary role for Notch signaling in alveolar formation through its action on the surrounding mesenchymal compartment (e.g. myofibroblasts), *Shh-Cre*-directed deletion of *Pofut1* or *Notch2* results in alveolar defects leading to postnatal emphysema-like enlargement of distal airspaces [7]. Evidently, *Notch2* is critical to the maintenance and/or proliferation of the AT2 cell population and its ablation inevitably results in a significant decrease in AT2 cells in the adult lung epithelium, and while conditional *Notch2* knockout induced failure to generate alveoli and *Notch1* knockout did not cause any notable morphological defects in lung development, *Notch1* and *Notch2* double knockout murine lung displayed a more severe phenotype than *Notch2* knockout alone, raising thus the possibility of a cooperative relationship between the two receptors in alveologenesis [7]. Of note, *Jag1* deletion in the distal epithelium results in a similar phenotype to conditional *Notch2* deficiency [45]. Clearly, further work is necessary to precisely define the cell-specific functions of Notch receptors and ligands as well as for the delineation of the cell-autonomous versus non-cell-autonomous effects.

## 3. Notch and NSCLC

Lung cancer is a heterogeneous disease driven by the presence of cancer stem cells (CSCs), also referred to as tumor-initiating cells. As one of the deadliest carcinomas, lung cancer accounts for 25% of all cancer related deaths [59]. NSCLC and small cell lung cancer (SCLC) are the two major subtypes of lung cancer, with NSCLC accounting for approximately 80–85% of all lung cancer cases [60]. NSCLC is further characterized into two major histological types: adenocarcinoma of the lung (ACL) and squamous cell carcinoma (SCC), and, by convention, differ in the location where they occur and the physical manifestations of the tumors. ACL generally occurs in distal airway and air exchange structures, in secretory cells, and is more common in non-smokers. SCC more commonly develops in the proximal region of the lung and has greater prevalence amongst smokers [61]. From a cell ontogeny standpoint, both ACL and SCC have been shown to originate from both club and AT2 cells [62,63,64,65,66], whereas basal cells are considered to also contribute to SCC development [67,68] and neuroendocrine cells have been ascribed as putative cells-of-origin of SCLC [69,70,71,72] (Figure 2).

The seminal discovery that Notch could be a catalyst in the onset of NSCLC came to light 20 years ago, when a somatic chromosomal translocation t(15;19) causing overexpression of the *Notch3* gene was discovered in poorly differentiated and aggressive lung adenocarcinoma [10]. Notably, in these studies, detailed karyotypic and molecular analysis on 44 cell lines, including the one where the t(15;19) genetic alteration was initially identified, demonstrated that 6 out of 7 cell lines where *Notch3* was found overexpressed indeed carried chromosome 19 translocations [10]. Importantly, the oncogenic properties of Notch3 have been further confirmed in studies demonstrating that a truncated, dominant negative form of Notch3 reduces tumor growth of t(15;19) NSCLC cell lines and that elevated expression of Notch3 occurs in 30–40% of primary lung tumors [11]. Furthermore, elevated expression of Notch3 in NSCLC has been seen associated with poor disease outcome [12], and NSCLC cell-derived xenografts treated with GSI MRK-003 displayed marked absence of Notch3^ICD^, which initially was significantly overexpressed, as well as remarkable decreased tumor growth capacity [73]. Considering that Notch3 seems to be the receptor most functionally-relevant in NSCLC tumors and cell lines, it is plausible to infer that the active form of Notch3 is necessary to NSCLC CSC maintenance, potency, and differentiation. In line with this concept, Notch3 has been recognized as a pivotal driver required for survival and maintenance of NSCLC CSCs both in humans and mice [74], and recent findings indicate that *Notch3* silencing inhibits EMT, decreases tumor cell proliferation, and induces apoptosis in NSCLC cells [14]. Markedly, these important findings are also supported by studies showing that elevated Notch activity can distinguish tumorsphere forming-competent NSCLC cells, and that a single cell of this high Notch population group can generate tumors and be able to self-renew [75]. Furthermore, there is evidence from signaling dynamics modeling and observations in breast cancer cells that suggests that Jagged-induced Notch activity may cause pushing circulating tumor cells to a survival-advantageous, epithelial/mesenchymal hybrid state [76], which, while it has been observed in NSCLC cells, it has, intriguingly, found to be destabilized and pushed towards full EMT upon Numb or Numbl silencing in hybrid state H1975 cells, thus indicating that Numb may act as a brake/modulating factor in EMT [77]. Of note, both Numb and Numbl act as molecular rheostats for Notch, and loss of Numb has been observed in 30% of NSCLC cases, where it furthermore serves as an indication of poor prognosis, generally associated with poor overall survival [77,78]. Together, these observations provide convincing evidence that Notch is responsible not only for NSCLC initiation, but also for the EMT and metastatic progression observed in patients with therapy resistance and tumor recurrence, and, thus, supports the notion that targeting Notch may be key in treating NSCLC tumors.

*Notch2* is found expressed at about 40% greater levels in patients with advanced stages of NSCLC compared to ones in stage I, and *Notch2* overexpression (22% increase) is seen to occur highly significantly in patients with disease recurrence [16]. Interestingly, Notch2 overexpression has also been consistently seen in invasive versus lepidic ACL cells, and this upregulation markedly coincided with expression upregulation of its putative downstream target, homeodomain transcription factor Six1, which, furthermore, together with Notch2, was shown to modulate EMT and the expression of *Smad3*, *Smad4*, and *Vimentin* in ACL cells [79]. Critically, a recent study on NSCLC chemosensitivity and CSCs has shown that ectopic expression of microRNA miR-181b suppresses CSC-like characteristics including tumorsphere formation and tumorigenecity in vitro and in xenograft models, and that these effects were mediated through downregulation of Notch2 transcription via interference binding to its 3′-UTR [80]. Notably, these studies also further confirmed that an inverse miR-181b and Notch2 expression relation is present in NSCLC tumors, and that Notch2 expression inversely correlated with both disease free survival (DFS) and overall survival (OS) in patients with disease stage I-II but not in advanced disease cases [80]. Intriguingly, a recent study in 2437 NSCLC tumor samples found that higher levels of *Notch1*, *Notch2*, *Jag1*, and *Dll1* mRNA were associated with better OS in ACL patients, while elevated *Notch3*, *Jag2* and *Dll3* mRNAs correlated with poor survival [81], and a report by Baumgart et al. showed that 67% of NSCLC tumors have either no or weak expression of Notch2 compared to normal lung tissue [82]. Noticeably, this latter work also disclosed that unlike Notch1 or Notch3, which displayed increased levels, Notch2 was decreased in advanced tumors in the *Kras^G12D^* NSCLC model, where it furthermore displayed a tumor suppressor function through modulation of the Wnt/β-catenin pathway [82]. Notch2 is the predominant receptor activated in AT2 cells and is required for their proliferation and maturation [7], and absence of the Wnt-downstream target Dlk1 (delta-like 1 homolog)—which also participates in lung development and inhibits endogenous Notch signaling—in AT2 cells during AT2 to AT1 repair-induced transition causes Notch activity upregulation and results in a stalled differentiation of AT2 to AT1 cells as well as in accumulation of an intermediate cell type [83]. While the observations above are discernibly contrasting in regard to an oncogenic role for Notch2 in NSCLC initiation and progression, the inductive exertion of transition states in AT2 cells [83] and its implication in EMT [79], as well as its predominant function in AT2 cell maturation and differentiation [7], and the fact that AT2 cells have been shown to functionally serve as cells-of-origin of NSCLC [63,84] (Figure 2), strongly warrants further exploration into the cell-specific, gain- and loss-of-function effects of this receptor in postnatal lung function and oncogenesis.

In T cell acute lymphoblastic leukemia (T-ALL), a translocation on chromosome 9 results in the generation of a truncated form of Notch1, which lacks its N-terminal domain and results in expression of the constitutively active form (Notch1^ICD^) of the receptor leading to malignant conversion [85]. In NSCLC, similarly to in T-ALL, activating mutations in the *Notch1* gene were found in 10% of NSCLC cases, and the presence of these alterations, which occur in the heterodimerization and PEST domains, were associated with worse prognosis in NSCLC patients proficient for the tumor suppressor p53 [78]. Notably, targeted expression of Notch1^ICD^ in the distal lung epithelium causes alveolar hyperplasia and adenoma formation, and a mixed manifestation of adenomas and adenocarcinomas is observed upon Myc cooperative action [13]. Interestingly, the Kras mutational activation effect that is exerted by Myc overexpression [86] was not observed in adenocarcinoma tumors resulting from Notch1^ICD^/Myc cooperation [13], clearly indicating that Notch1 can substitute for Kras activation in Myc-driven tumorigenesis. Of marked importance, it has been determined that Kras-triggered murine NSCLC tumors display significantly higher levels of Notch1^ICD^ and Hes1, which furthermore is associated with tumor grade [87], thus directly suggesting that, in the context of Kras-driven NSCLC, Notch may act as downstream mediator and, plausibly, as an actionable target. Critically, genetic interaction studies have recently demonstrated that Notch1 activation is indeed required for Kras-triggered lung adenocarcinoma formation and NSCLC regulation of cell survival through modulation of p53 [88], and it has been shown that pharmacological inhibition of Notch signaling with GSI LY411575 suppresses Kras-driven lung adenocarcinoma tumor growth [87]. Another important aspect of Notch1 action in NSCLC is in respect to oxygen modulation, treatment susceptibility and CSCs. In ACL, hypoxia-inducible factor-1α (HIF-1α) controls Notch1 activity under hypoxia, whereas Notch1^ICD^ is undetectable in non-hypoxic regions of tumors [89], critical findings that are related to what has been observed in T-ALL cells where Notch1 expression and activation, as well as mRNA and protein levels of its downstream target Hes1, are upregulated under hypoxic conditions [90]. Notably, in both ACL and T-ALL cells, HIF-1α has been shown to functionally act as an upstream positive regulator of Notch1 expression and action, and Notch1 has been ascribed as a molecular transducer for HIF-1α proliferative, pro-survival, and chemoresistance signals [89,90]. Remarkably, IGF1R (insulin-like growth factor 1 receptor) mRNA and protein expression levels are directly correlated with shorter PFS and OS in NSCLC patients either with or without association to EGFR (epidermal growth factor receptor) [91,92], and Notch1 has been found to form a transcriptional complex in an Rbpjk-recognition motif located in intron 1 of *IGFR* and regulate its expression, along with Akt1 phosphorylation, downstream of HIF-1α [89], further confirming Notch1’s role as a main transducer hub for HIF-1α/Akt-1/IGFR molecular circuitry. Notably, Notch1, HIF-1α, and IGFR have been shown to be implicated in NSCLC EMT and CSC survival and self-renewal [93,94,95], and Notch1 has been found highly expressed in CD44^+^CD24^−^ A549 CSCs, where its inhibition, through either siRNA or DAPT, markedly reduced colony-forming capacity [93]. Like CD44, ALDH1 (aldehyde dehydrogenase 1) has also been ascribed as a marker for CSCs in NSCLC [96], where, remarkably, mRNA levels for *Notch1*, *Notch2*, *Notch3*, and their downstream effectors *Hes1*, *Hey1*, and *Hey2* are found significantly upregulated compared to ALDH^−^ cells [97]. Noticeably, in this latter study, it was further found that shRNA-mediated silencing of *Notch3* in H358 and H2009 NSCLC cell lines resulted in lower levels of *Hey1* and *Hey2* along with decreased clonogenic capacity and reduced number of ALDH^+^ cells [97]. Interestingly, in the *Kras^G12D^;Trp53^flox/flox^;eYFP* murine model of NSCLC, a putative CSC population with augmented sphere forming ability was identified as markedly positive for CD24, ITGB4 (integrin subunit beta 4), and Notch1, Notch2 and Notch4 (CD24^+^ITGB4^+^Notch^hi^), and furthermore, actionably relevant in chemoresistance acquisition and functionally dependent on Notch3 action [74]. Several prospective CSC populations have been identified in human and murine NSCLC through the use of a number of distinct markers and/or a combination of them, and while the identity of these cells seems, at present, quite diverse, Notch has been observed as a common denominator in this cell entity, not only as a marker for their profiling and identification, but also, and more importantly, functionally.

Mutational status in Notch signaling and NSCLC indicates that this pathway’s components are frequently mutated in this pathology, although, in some instances, with converse mutations with regard to their ability to aberrantly hyperactivate the pathway or impair signaling activity (Table 1). Identified *Notch1* genetic alterations are presumed to be activating and are reported to include somatic frame shift (fs) mutations in the TAD and PEST domains (S2275fs and V2444fs, respectively) and non-synonymous substitutions in the heterodimerization region and TAD domain (D1643H and R2328W, respectively), and, importantly, are demonstrated to be susceptible to inhibition by GSIs DAPT and MRK-003 and, furthermore, required in driving sustained tumor cell survival [78], thus providing a rationalized framework for the devising of Notch1-targeting therapies. *Notch2* alterations in NSCLC, on the other hand, have been reported to include both activating and inactivating mutations, and, in contrast to what has been observed in *Notch1*, these alterations are mainly found to be gene amplifications (TCGA, MSKCC, cBioportal (www.cbioportal.org [98,99])), both in ACL and SCC, which could in fact go along with the increased expression observed in patients with advanced NSCLC stage and disease relapse [16]. Notably, *Rumi* (*Poglut1*) amplification has also been observed in NSCLC patients, and the protein levels of this Notch regulator in NSCLC tumors are directly associated with poor prognosis and decreased patient survival [100]. Although the specific effects of the distinct *Notch2* point mutations have not been functionally experimentally tested yet, this is a subject that reclaims further exploration to discern Notch2 functional action in NSCLC. Apart from the initial findings on t(15;19) translocations involving aberrant activation of Notch3 in NSCLCs, the activity of this receptor has also been found required during treatment refractoriness evolution in EGFR mutant NSCLC [101]. Interestingly, in this latter phenomenon, the observed tumor driving induction effect of Notch3 seems to be dependent on direct physical (biochemical) interaction with EGFR and Notch3 tyrosine phosphorylation modification, although the exact residue(s) undergoing this posttranslational regulation has not yet been identified [101]. Remarkably, it should be noted that while Notch3 mediates EGFR regulation of the Wnt/β-catenin pathway in EGFR mutant NSCLC, a dominant negative form of Mastermind-like (dn-MAML) is ineffective in preventing the expansion of ALDH^+^ CSCs in these tumors, indicating that Notch3 action is likely due to non-canonical activity [102]. Notably, in Kras-mutant ACL tumor cells upregulation of Notch3 expression has also been observed in a signaling pathways’ interplay requiring PKCι-mediated ELF3 phosphorylation and the subsequent *Notch3* promoter occupancy and transcriptional activation that, furthermore, leads to enhanced CSC phenotype [103]. In addition, it has recently been reported that C381T and G684A synonymous polymorphisms in the *Notch3* gene (Table 1) are significantly linked to increased lung cancer development susceptibility [104,105]. All these observations would thus indicate that not only is Notch3 a key factor in NSCLC development, but that its genetic and epigenetic regulation is central in treatment resistance and disease progression. Importantly, the findings described above are in marked contrast to what occurs in SCLC, where Notch, likely as a developmental evolutionary reminiscence, plays a tumor suppressive role, as evidenced by the presence of inactivating mutations in Notch components in 25% of SCLC tumors and the high levels of both the non-canonical Notch inhibitor, Dlk1, and the neuroendocrine-oncogenic factor, and Notch negatively-regulated gene, Ascl1 [106]. Interestingly in SCLC cells, histone H3K27 acetylation of *Notch1* promoter and its transcriptional activation occurs when they are subjected to LSD1 (lysine-specific histone demethylase 1A) inhibition, in an event that further conduces to repression of Ascl1 expression and reduced tumor cell viability [107]. Although neither germ-line nor somatic mutations in Notch4 have been systematically explored in NSCLC, a P1663Q mutation (Table 1) in the ankyrin repeat (ANK) region of the receptor (preliminarily predicted to act as an oncogenic, gain-of-function alteration) has been identified in Hispanic ACL patients [108], and, furthermore, a homogeneous (presumably non-somatic) mutation has been reported in malignant pulmonary granular cell tumor [109]. Notch ligands’ mutations have been less well studied/characterized in NSCLC primary tumors, although they have been found in various NSCLC cell lines, albeit with a relatively lower frequency than in Notch receptor genes [110], and the expression levels of these Notch non-cell autonomous factors have been observed to be consistently lower in NSCLC tumors compared to surrounding, non cancerous tissue, with increased Jag1 levels among NSCLC tumors serving, nonetheless, as an indicator of poor overall survival [111]. Collectively, the findings above clearly indicate that not only somatic alterations are involved in Notch activity in lung cancers, but also that the developmental programs used in Notch-regulated fate choices and epigenetic modulation are key in both SCLC and NSCLC.

An unexplained oncogenic manifestation noted in NSCLC is the existence of a hybrid histopathological subtype harboring hallmarks of both ACL and SCC [112,113]. In tumors with this unique phenotypic presentation, a genetic interaction was noted between deficiency of tumor suppressor Lkb1 (Stk11) and Kras activation [114,115]. Mechanistically, Lkb1 behaves as a molecular rheostat for Kras, and in the context of Lkb1 deficiency, Kras aberrant activation has been found to induce the formation of both lung adenosquamous cell carcinoma and SCC [115]. It is worth noting, in this respect, that activating mutations of Kras are capable of giving rise to adenocarcinoma and no other subtype of NSCLC [116]. The fact that the loss of Lkb1 alone does not induce lung tumor formation [115] indicates the existence of a critical mutational cooperation where concomitant Kras activation and Lkb1 loss drive the formation of adenosquamous cell carcinoma and SCC. Interestingly, the Kras and Lkb1 cooperative mutational synergism has also been shown to be critical, and plausibly causative, of PD-1/PD-L1 inhibition resistance [117], and Notch inactivation has been established as epistatic to oncogenic Kras activation in triggering malignant transformation in the pulmonary epithelium [87,88], making it thus discernibly reasonable to infer that Notch inhibition could actually have a suppressive effect on the Kras/Lkb1 mutational cooperation, not only in the development of adenosquamous cell carcinoma and squamous cell carcinoma, but also in NSCLC metastatic progression and treatment resistance acquisition. Evidently adding more complexity to previous findings indicating a proto-oncogenic role of Notch in NSCLC tumors is the recent discovery that Lkb1 deficiency in A549 cells sustains the overexpression of cAMP–regulated transcriptional coactivator 2 (CRTC2), stimulating the upregulation of the oncogene inhibitor of DNA binding 1 (ID1) [118]. Notably, ID1, known to contribute to tumorigenesis in several carcinomas [119,120], has been found to interact with Notch downstream effector and molecular oscillator Hes1 during neurogenesis [76,121] and—markedly intriguingly—also attenuate Notch signaling activity via Deltex1 upregulation in Notch-mediated differentiation of T-cells [122]. Along, these observations would seem to suggest that Lkb1 loss, via activation of the ID1 pathway, would interact with Notch in NSCLC tumors and, therefore, would render Notch activity functionally relevant in the development and progression of tumors of this subtype. In line to these findings, nonetheless, is the observation that Notch action inhibition in H1299 and A549 Kras mutant cells with γ-secretase inhibitor DAPT, alone or in combination with gemcitabine, had a marked effect in NSCLC cell viability and colony-forming potential [123], and that Rumi knockdown, globally inactivating Notch signaling in NSCLC A549 and H23 cells (both Lkb1-deficient [118,124]) results in marked anti-oncogenic exertion, including a significant reduction in cell proliferation, migration and survival [100]. These concurrent findings, therefore, would positively suggest that in the context of Lkb1 deficiency Notch is also in fact promoting tumor growth and neoplastic progression, and that it is possible that ID1 may be a cooperative factor likely releasing the otherwise oscillatory negative autoregulation of Hes1, and, therefore, further indicating that the molecular interaction between these two pathways is, in this case, indispensable. Furthermore, and adding also more support to this respect, it is interesting to note that high levels of ID1 act as a biomarker in Kras mutant adenocarcinomas and predict poor survival outcome [125]. Clearly, further studies steered towards exploring Notch gain- and loss-of-function activity in the context of hyper-activated and inactivated ID1 would confirm the Lkb1/ID1/Notch molecular wiring in adenosquamous cell carcinoma formation and NSCLC progression, metastatic dissemination and therapy resistance.

## 4. Therapy Perspectives

**Figure 3:**
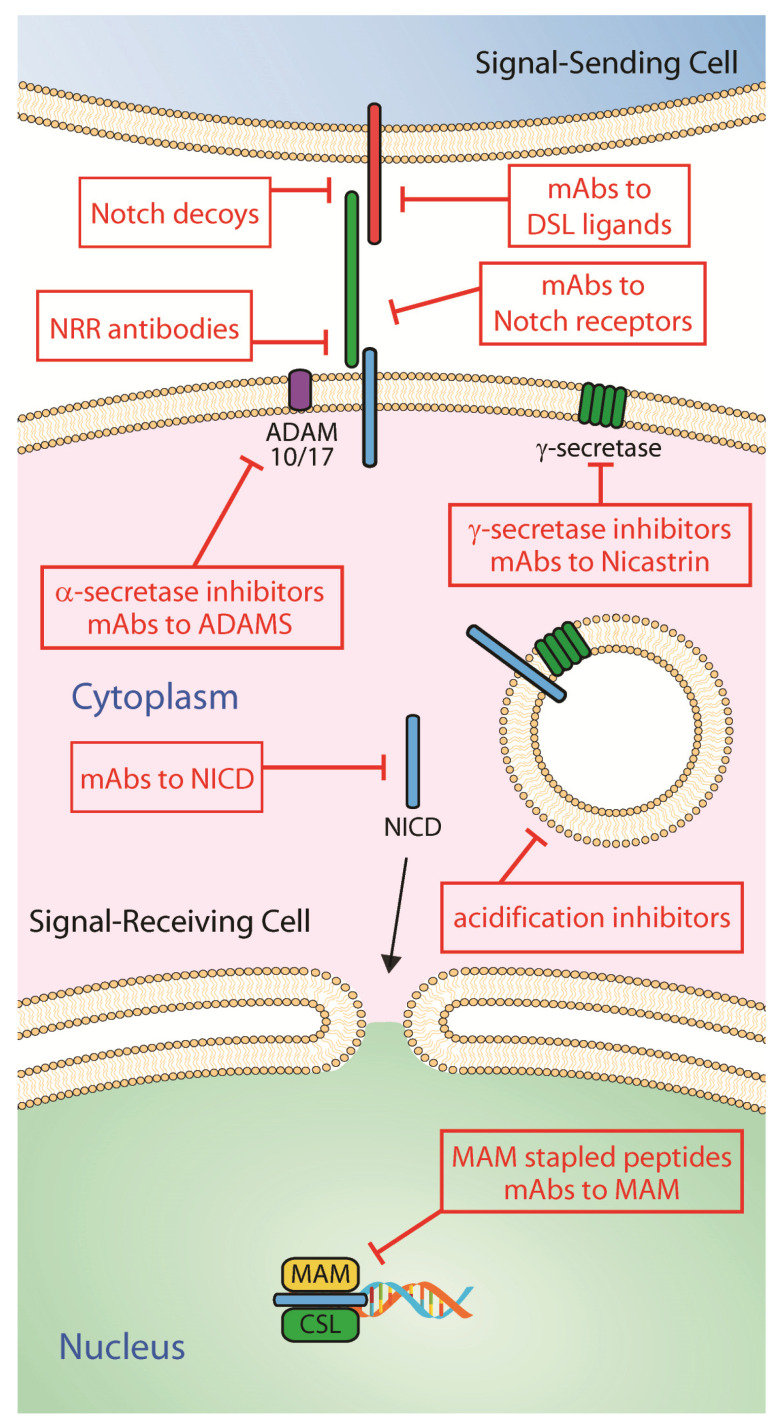
Therapeutic strategies to modulate Notch signaling activity. Potential therapeutic inhibitors to block Notch signaling activity include soluble decoy receptors, monoclonal antibodies (mAbs) to Notch receptors and mAbs to DSL ligands to disrupt/prevent receptor/ligand binding interaction; antibodies recognizing Notch proteins’ NRR domain, mAbs to ADAMS and α-secretase inhibitors to block S2 proteolytical processing; γ-secretase small molecule inhibitors and mAbs to Nicastrin to prevent S3 cleavage and NICD production; endosome acidification inhibitors to halt endosomal NICD release; blocking mAbs to inactivate NICD; and stapled peptides and mAbs to MAM to disrupt MAM/NICD/CSL complexes.

The existence of distinct mutations in NSCLC creates complexity in establishing a treatment effective for all cases of NSCLC. Oncogenic driver mutations commonly found in NSCLC include EGFR, ALK, DDR1, Kras and Notch, each contributing to the therapeutic outcome/resistance observed when administered conventional and/or targeted therapy [2,125,126]. In several contexts, Notch has been reported to be found irregularly expressed and/or mutated, and it has been found to synergize with other mutations to create a more severe phenotype exhibiting greater therapeutic resistance [127]. In order to better understand the relationship between Notch and other oncogenic driver mechanisms in NSCLC, a number of studies have been proposed and conducted in which Notch activity is manipulated through various modulatory strategies including, among others, GSIs, blocking monoclonal antibodies against Notch receptors and ligands, alpha secretase inhibitors to target ADAMs, and stapled peptides to block Notch/Mastermind interaction (Figure 3 and Table 2). Some of these modalities have been explored in NSCLC while others have been tested in various types of malignancies, including solid tumors, and in animal models of human cancers [128,129,130].

Inhibition of the Notch pathway with GSI LSN-411575 has been proven to block NSCLC tumor growth in vivo in Kras murine models, where, in addition to inducing Hes1 downregulation, it affects ERK phosphorylation, inhibits cell proliferation and increases apoptosis [87]. Furthermore, DAPT treatment has been shown effective in more pronouncedly decrease viability of putative CD133^+^ CSCs derived from A549 cells compared to CD133^−^ cells, and significantly increase the proportion of CD133^+^ cells in cell cycle S phase [131]. In addition, another GSI, MRK-003 has been tested in xenograft models and been shown efficacious in reducing tumor growth through cell death induction via downregulation of levels of anti-apoptotic phospho-Bcl-2 and Bcl-xL [73], while monotherapy with GSI BMS-906024 had a significant (*p* < 0.05) specific spheroid growth delay in 3D spheroid forming assays [132]. Also as single agents, MRK-003 and DAPT were tested in primary NSCLC cultures and shown to be effective in decreasing growth potential and cell survival in both Numb-Low and Notch1-mutant cells [78], while 50 μM DAPT treatment for two weeks in high-Notch CD44^+^CD24^−^ CSCs results in reduced cell growth and decreased colony-forming ability [93]. While the studies above described are very encouraging in regard to ascribing Notch as a druggable actionable target in NSCLC, it should be, however, noted that despite being quite effective in blocking Notch activity, GSIs are markedly notorious for their adverse side effects, which include mainly diarrhea and gastrointestinal complications [130]. This undesirable toxicity is likely caused by Notch “on-target” effects due to the heightened Notch activity present in gastrointestinal precursor cells and, also plausible but to a lesser extent, Notch “off-target” effects due to the wide proteolytic processing capacity of the gamma secretase complex (about 90 targets) [133]. Interestingly, a number of research studies have also recently explored the combined use of GSIs along with chemotherapy and targeted therapies [131,134,135,136,137], which moreover further provide a strong rationale for devising synthetic lethality-based therapies [138]. Liu et al. noted that when A549 cells were concomitantly treated with DAPT and cisplatin, a marked synergistic effect was observed in cell viability in both CD133^−^ and CD133^+^ cells, and that this combination therapy also significantly induced cell cycle G2/M phase arrest in CD133^+^ cells [131]. Markedly, BMS-906024 has also been shown to enhance the antitumor activity of paclitaxel and cisplatin in NSCLC cell lines, and a marked synergy was observed only between paclitaxel and BMS-906024 in patient-derived xenograft (PDX) models wild-type for Notch through increased apoptosis and decreased cell proliferation, but in a p21- and p57-independent fashion [135]. Interestingly, the co-inhibition of Notch and EGFR pathways has also been explored, particularly in the context of EGFR tyrosine kinase inhibitor (TKI) acquired resistance [134,136]. Gain-of-function EGFR mutations are the most frequent alterations observed in NSCLC, occurring in approximately 27% of NSCLC cases [139], and while targeted therapies are available for these tumor types, most patients who initially benefit from EGFR-TKIs eventually develop resistance to these agents, likely as a result of secondary genetic alterations [140]. In two separate studies in NSCLC xenograft models, Xie et al. showed that DAPT and BMS-708163 can overcome resistance to EGFR-TKI gefitinib [134,136]. Thus, combined gefitinib-DAPT treatment of Balb/c nu/nu mice with EGFR-TKI acquired resistant NSCLC xenografts resulted in marked tumor growth retardation, decreased proliferative activity, and increased apoptotic cell death accompanied by upregulation of active Caspase-3 and EMT phenotype attenuation [134], while co-administration of BMS-708163 and gefitinib resulted in marked reduction of tumor size, increased levels of Caspase-3, and decreased expression of Ki-67 [136]. Importantly, concomitant inhibition of Notch and DDR1 (discoidin domain receptor 1) with GSI LY411575 and 7rh, a benzamide TKI, in Kras-driven murine tumors had an additive effect on apoptosis induction and tumor growth blockage in both p53-proficiency and p53-deficiency contexts, and the co-inhibition of these two pathways with demcizumab (Dll4 blocking antibody) and dasatinib (TKI) markedly reduced tumor growth in Kras-mutant PDX models in a manner comparable to standard cisplatin/paclitaxel chemotherapy [137].

Although not extensively explored, endosome acidification inhibition is another attractive approach to block Notch signaling activity [141,142,143]. Endocytosis of Notch receptors in the signal-receiving cell is currently considered also a critical step in signaling modulation, downregulating Notch receptors that have not been activated through ligand binding-mediated proteolysis and, in aberrant contexts, abnormally triggering Notch cleavage through increased gamma secretase activity due to endosome acidification [141,142,143,144,145]. This mechanism of Notch activation, particularly relevant in scenarios of ligand-independent signaling, has been well studied in *Drosophila melanogaster* where a variety of studies with mutants in the endosomal trafficking machinery and apico-basal polarity complexes, including *lgd* (*lethal (2) giant discs 1*), *lgl* (*lethal (2) giant larvae*), *scrib* (*scribble*) and *dlg* (*discs large*), have shown that endosomal acidification induces ligand-independent, gamma secretase-mediated Notch hyperactivation [146,147,148,149,150,151,152]. In this regard, recent studies have demonstrated that proton pump V-ATPase (vacuolar adenosine triphosphatase) and Vap33 (vesicle associate protein, 33kDa) rheostatic interaction is modulated by lgl in endosome acidification control, and that when this mechanism is disrupted upon loss of lgl, endosome acidification and aberrant Notch activation are observed [146]. Along with this, pharmacological blockade of V-ATPase with bafilomycinA1 (BafA1) in *Drosophila* and zebrafish developing tissues, as well as in normal breast cells and Notch-addicted breast cancer cells results in decreased Notch processing and signaling activity [153]. Interestingly, a recent study by Pinazza et al. has shown that lysosome inhibitors chloroquine and BafA1 were able to rescue the reduced Notch3 levels observed upon HDAC (hystone deactylase) inhibition with Trichostatin A in T-ALL cells [154]. Although these observations are contrasting with regard to the specific Notch degradation mechanism along the endocytic route and lysosome compartment, and further studies are thus obviously required, HDAC6 inhibition through tubacin and its genetic silencing in T-ALL PDX models demonstrated an interplay between HDAC6 and the endocytic/lysosomal pathway and, furthermore, the antineoplastic effects of HDAC6 blockage [154]. While not explored yet in NSCLC, endocytic/lysosomal acidification inhibition approaches as well as the dissecting of other Notch-regulating molecular mechanisms able to modulate its strength and block aberrant, ligand-independent Notch activity [155,156] are a subject of much urgency and critical importance, as this mode of Notch signaling activation is discernibly primarily involved in neoplastic cell growth.

Clinical studies targeting Notch have been initiated since a few years ago and have been mainly based in the use of GSIs, although, more recently, monoclonal antibodies against Notch receptors and ligands have been developed (Table 2). LY3039478 is a potent GSI that has demonstrated antitumor activity in xenograft models, including lung cancer [157], and in patients with distinct neoplasias [158,159], and clinical trials are currently undergoing or have just been completed in patients with advanced solid tumors and with gene/protein alterations related to the Notch pathway (NCT02836600, NCT02784795). Another GSI, RO4929097, has significant activity in blocking Notch processing and activity, and is efficacious in markedly inducing tumor growth inhibition in NSCLC xenografts [160]. This inhibitor has been tested alone in NSCLC patients (NCT01193868) or in combination with agents targeting VEGFR (cediranib) or mTOR (temsirolimus) pathways in patients with solid tumors [161,162]. In combinational regimens, the use of this GSI with cediranib and temsirolimus had a slight improved outcome with 58% and 73% of the patients displaying stable disease, respectively [161,162], while the Phase 2 trial with NSCLC patients had to be prematurely terminated due to a drug production halt. Eli Lilly’s LY900009 is another GSI that has been tested in patients with various solid tumors including NSCLC [163]. In these studies, 5 out of 35 patients (14%) displayed stable disease and the Notch "on-target" effect was confirmed [163]. Notably, a Phase 1 study with GSI PF-03084014 in 64 patients with solid tumors, and including patients with lung cancer, showed an objective response rate of 13% among 46 response-evaluable patients [164], and in 29% of patients with desmoid tumors treated with this GSI partial response was observed [165]. While robust effects with GSIs have not yet been achieved, it should be considered that several factors may affect reaching more meaningful conclusions in this respect. In general, patients have not been systematically pre-screened for the presence of Notch activating mutations in the described studies, and the indicated GSIs also differ in their intrinsic inhibitory properties and "off-target" effects. Furthermore, some of the studies specifically involving NSCLC patients have not been completed and/or were prematurely halted. While GSI-based strategies still need further investigation, another modality that has been more recently developed to attain Notch inhibition in cancer tumors is through the use of antibodies and antibody-based derivatives (Table 2). In this context, demcizumab, an IgG2 humanized monoclonal antibody directed against Dll4, was initially tested in combinational therapies with pemetrexed and carboplatin in non-squamous NSCLC, where 20 out of 40 patients had objective tumor response [166], although, unfortunately however, showed no benefit in a more recent, completed Phase 2 study (NCT02259582). Rovalpituzumab tesirine, an antibody-drug conjugate targeting Dll3, was tested in SCLC patients with initial encouraging results, with 18% of patients displaying objective response [167], but it however showed only modest benefits in Phase 2 studies [168]. Also in SCLC patients, a combinational regime of tarextumab (a Notch2/3 antibody) with chemotherapy, failed to improve progression free survival or overall survival in a recent Phase 2 clinical trial [169]. Notably, enoticumab, a fully human IgG1 monoclonal antibody that binds human Dll4, has been recently tested in human patients with advanced solid tumors, and demonstrated effective monotherapy clinical activity, as indicated by the observed prolonged stable disease in some of the cancers under study, and the partial response attained in patients with ovarian carcinoma and bronchioalveolar-type NSCLC [170]. It should be noted, though, that while immunotherapies with tarextumab and rovalpituzumab have not provided the desired outcomes in SCLC, and that it is plausible that Notch-induced intratumor heterogeneity [171] may partly be responsible for the inefficacious results with these targeting agents, loss-of-function Notch mutations and Notch overexpression studies [106,172] have collectively indicated that this pathway is rather tumor-suppressive in this pathology. Thus, from a developmental and genetic perspective, and considering that Notch exerts a neurogenic suppressor function during neuroendocrine/non-neuroendocrine fate choice, the failure of Notch inhibition in SCLC would actually come as not too surprising. From this standpoint, the efficacious action of enoticumab in bronchioalveolar NSCLC should, therefore, be actually appreciated also in light to the developmental programs deployed during lung morphogenesis and ostensibly conserved in lung oncogenesis. More work in this realm is definitively needed as Notch remains a very attractive actionable target in NSCLC.

*Anaplastic lymphoma kinase* (*ALK*) is a tyrosine kinase receptor gene susceptible to fusing with *echinoderm microtubule-associated protein-like 4* (*EML4*), resulting in NSCLC harboring the *EML4-ALK* oncogene [173]. Alterations in the ALK pathway are observed in approximately 5% of cases of NSCLC in a manner mutually exclusive with Kras and EGFR mutations [174]. Similar to EGFR, while ALK inhibitors are effective in initially treating tumors harboring the *EML4-ALK* mutation, secondary *ALK* mutations and EMT desensitize patients to ALK targeted treatment [175], and the acquired resistance to next generation ALK inhibitors in NSCLC is most common in hypoxia via induction of EMT [175]. Given that Notch has been found to have a critical role in EMT [76] and tumor hypoxia signaling crosstalk [89,90], it stands to reason to infer that the inhibition of Notch may sensitize EML4-ALK TKI resistant NSCLCs. While this idea has not been exhaustively tested, a recent report from the Vooijs laboratory has reported that the combination of GSI BMS-906024 and crizotinib (EML4-ALK TKI) resulted in a significant delay in spheroid growth in H1299 and H460 cells, which was further more pronounced by inclusion of radiation [132]. Importantly, these observations are also supported by recent studies in anaplastic large cell lymphoma, where treatment of GSIs Z-LLNIe-CHO (GSI-I) and PF-0308414 with crizotinib led to additive to synergistic effects in reduction of cell proliferation and apoptosis induction in these tumor type cells [176].

Amgen’s AMG510 and Mirati’s MTRX849 are two recently developed Kras^G12C^ inhibitors that have been markedly shown effective in inducing NSCLC tumor regression in xenograft-bearing mice and patients [177,178]. Although these novel compounds are quite encouraging and offer new hope for NSCLC patients carrying the *Kras^G12C^* mutation, Kras gain-of-function mutations in NSCLC also include *Kras^G12D^* and *Kras^G12V^* [179,180], which are either not or less preferentially targeted by the above-mentioned compounds [177,178]. In this respect, it is important to mention that pharmacological or genetic inactivation of Notch alone has been shown to have an epistatic effect to both *Kras^G12D^* and *Kras^G12V^* mutations [87,88]. Furthermore, genetic silencing of Rumi, which regulates both ligand-dependent and -independent Notch activity [155,156], caused marked cell proliferation decrease and cell cycle S-phase arrest in both Kras^G12S^ A549 cells and Kras^G12C^ H23 cells [100].

From its discovery by John Dexter over a century ago [181], the keen interest of Morgan and its allelic series [182,183], the uncovering of its neurogenic suppression properties by Poulson [184], the positional cloning of the *Drosophila*’s receptor as a transmembrane glycoprotein transducer by Artavanis-Tsakonas and Young [185,186], and the disclosing of the quantum mechanics-based, ligand-induced activation [20], the Notch pathway has revealed a versatile cell communication logic serving critical roles in morphogenesis and disease, including lung cancer. Clearly, further explorations into the mechanistic aspects of its regulation and cellular and molecular dynamics promise to provide an in-depth insight into its inductive and instructive roles in animal development and precision cancer medicine.

## Figures and Tables

**Table 1:** Mutational status of Notch receptors in NSCLC.

Receptor	Genetic Alteration
Notch1	S2275fs (activating) V2444fs (activating) D1643H (activating) R2328W (activating)
Notch2	Gene amplification (activating) GOF point mutations (activating) * LOF point mutations (inactivating) *
Notch3	Translocation (activating) C381T (DNA polymorphism, increased NSCLC susceptibility) G684A (DNA polymorphism, increased NSCLC susceptibility)
Notch4	P1663Q (activating)

* Multiple mutations have been found (cBioportal) and predicted to be either hyperactive gain-of-function (GOF) or inactivating loss-of-function (LOF). These point mutations have not been experimentally characterized yet.

**Table 2:** Main gamma secretase inhibitors and Notch targeting antibodies used in clinical and subclinical studies in lung development and cancer.

Gamma Secretase Inhibitor	Chemical Formula
DAPT	C_23_H_26_F_2_N_2_O_4_
PF-03084014	C_27_H_41_F_2_N_5_O
RO4929097	C_22_H_20_F_5_N_3_O_3_
LY3039478	C_22_H_23_F_3_N_4_O_4_
LY900009	C_23_H_27_N_3_O_4_
LY411575	C_26_H_23_F_2_N_3_O_4_
Z-LLNIe-CHO (GSI-I)	C_26_H_41_N_3_O_5_
MK-0752	C_21_H_21_ClF_2_O_4_S
MRK-003	C_25_H_31_F_6_N_3_O_2_S
BMS-906024	C_26_H_26_F_6_N_4_O_3_
BMS-708163	C_20_H_17_ClF_4_N_4_O_4_S
**Notch therapeutic antibody**	**Molecular target**
Enoticumab	Dll4
Demcizumab	Dll4
MEDI0639	Dll4
Rovalpituzumab tesirine	Dll3
Tarextumab	Notch2, Notch3
Jag1.b70	Jag1
Jag2.b33	Jag2

## References

[1] Duvic B., Hoffmann J.A., Meister M., Royet J. (2002). Notch signaling controls lineage specification during Drosophila larval hematopoiesis. Curr. Biol. CB.

[2] Zou B., Zhou X.L., Lai S.Q., Liu J.C. (2018). Notch signaling and non-small cell lung cancer. Oncol. Lett..

[3] Ito T., Udaka N., Yazawa T., Okudela K., Hayashi H., Sudo T., Guillemot F., Kageyama R., Kitamura H. (2000). Basic helix-loop-helix transcription factors regulate the neuroendocrine differentiation of fetal mouse pulmonary epithelium. Development.

[4] Guseh J.S., Bores S.A., Stanger B.Z., Zhou Q., Anderson W.J., Melton D.A., Rajagopal J. (2009). Notch signaling promotes airway mucous metaplasia and inhibits alveolar development. Development.

[5] Tsao P.N., Chen F., Izvolsky K.I., Walker J., Kukuruzinska M.A., Lu J., Cardoso W.V. (2008). Gamma-secretase activation of notch signaling regulates the balance of proximal and distal fates in progenitor cells of the developing lung. J. Biol. Chem..

[6] Tsao P.N., Vasconcelos M., Izvolsky K.I., Qian J., Lu J., Cardoso W.V. (2009). Notch signaling controls the balance of ciliated and secretory cell fates in developing airways. Development.

[7] Tsao P.N., Matsuoka C., Wei S.C., Sato A., Sato S., Hasegawa K., Chen H.K., Ling T.Y., Mori M., Cardoso W.V. (2016). Epithelial Notch signaling regulates lung alveolar morphogenesis and airway epithelial integrity. Proc. Natl. Acad. Sci. USA.

[8] Xu K., Nieuwenhuis E., Cohen B.L., Wang W., Canty A.J., Danska J.S., Coultas L., Rossant J., Wu M.Y., Piscione T.D. (2010). Lunatic Fringe-mediated Notch signaling is required for lung alveogenesis. Am. J. Physiol. Lung Cell. Mol. Physiol..

[9] Yao E., Lin C., Wu Q., Zhang K., Song H., Chuang P.T. (2018). Notch Signaling Controls Transdifferentiation of Pulmonary Neuroendocrine Cells in Response to Lung Injury. Stem Cells.

[10] Dang T.P., Gazdar A.F., Virmani A.K., Sepetavec T., Hande K.R., Minna J.D., Roberts J.R., Carbone D.P. (2000). Chromosome 19 translocation, overexpression of Notch3, and human lung cancer. J. Natl. Cancer Inst..

[11] Haruki N., Kawaguchi K.S., Eichenberger S., Massion P.P., Olson S., Gonzalez A., Carbone D.P., Dang T.P. (2005). Dominant-negative Notch3 receptor inhibits mitogen-activated protein kinase pathway and the growth of human lung cancers. Cancer Res..

[12] Ye Y.Z., Zhang Z.H., Fan X.Y., Xu X.L., Chen M.L., Chang B.W., Zhang Y.B. (2013). Notch3 overexpression associates with poor prognosis in human non-small-cell lung cancer. Med Oncol..

[13] Allen T.D., Rodriguez E.M., Jones K.D., Bishop J.M. (2011). Activated Notch1 induces lung adenomas in mice and cooperates with Myc in the generation of lung adenocarcinoma. Cancer Res..

[14] Hassan W.A., Yoshida R., Kudoh S., Motooka Y., Ito T. (2016). Evaluation of role of Notch3 signaling pathway in human lung cancer cells. J. Cancer Res. Clin. Oncol..

[15] Yuan X., Wu H., Xu H., Han N., Chu Q., Yu S., Chen Y., Wu K. (2015). Meta-analysis reveals the correlation of Notch signaling with non-small cell lung cancer progression and prognosis. Sci. Rep..

[16] Chen C.Y., Chen Y.Y., Hsieh M.S., Ho C.C., Chen K.Y., Shih J.Y., Yu C.J. (2017). Expression of Notch Gene and Its Impact on Survival of Patients with Resectable Non-small Cell Lung Cancer. J. Cancer.

[17] Donnem T., Andersen S., Al-Shibli K., Al-Saad S., Busund L.T., Bremnes R.M. (2010). Prognostic impact of Notch ligands and receptors in nonsmall cell lung cancer: Coexpression of Notch-1 and vascular endothelial growth factor-A predicts poor survival. Cancer.

[18] Kovall R.A., Gebelein B., Sprinzak D., Kopan R. (2017). The Canonical Notch Signaling Pathway: Structural and Biochemical Insights into Shape, Sugar, and Force. Dev. Cell.

[19] Kopan R., Ilagan M.X. (2009). The canonical Notch signaling pathway: Unfolding the activation mechanism. Cell.

[20] Luca V.C., Kim B.C., Ge C., Kakuda S., Wu D., Roein-Peikar M., Haltiwanger R.S., Zhu C., Ha T., Garcia K.C. (2017). Notch-Jagged complex structure implicates a catch bond in tuning ligand sensitivity. Science.

[21] Schittny J.C. (2017). Development of the lung. Cell Tissue Res..

[22] Cardoso W.V., Lu J. (2006). Regulation of early lung morphogenesis: Questions, facts and controversies. Development.

[23] Herriges M., Morrisey E.E. (2014). Lung development: Orchestrating the generation and regeneration of a complex organ. Development.

[24] Burri P.H. (1984). Fetal and postnatal development of the lung. Annu. Rev. Physiol..

[25] Jain R., Barkauskas C.E., Takeda N., Bowie E.J., Aghajanian H., Wang Q., Padmanabhan A., Manderfield L.J., Gupta M., Li D. (2015). Plasticity of Hopx(+) type I alveolar cells to regenerate type II cells in the lung. Nat. Commun..

[26] Desai T.J., Brownfield D.G., Krasnow M.A. (2014). Alveolar progenitor and stem cells in lung development, renewal and cancer. Nature.

[27] Treutlein B., Brownfield D.G., Wu A.R., Neff N.F., Mantalas G.L., Espinoza F.H., Desai T.J., Krasnow M.A., Quake S.R. (2014). Reconstructing lineage hierarchies of the distal lung epithelium using single-cell RNA-seq. Nature.

[28] Barkauskas C.E., Cronce M.J., Rackley C.R., Bowie E.J., Keene D.R., Stripp B.R., Randell S.H., Noble P.W., Hogan B.L. (2013). Type 2 alveolar cells are stem cells in adult lung. J. Clin. Investig..

[29] Wang Y., Tang Z., Huang H., Li J., Wang Z., Yu Y., Zhang C., Li J., Dai H., Wang F. (2018). Pulmonary alveolar type I cell population consists of two distinct subtypes that differ in cell fate. Proc. Natl. Acad. Sci. USA.

[30] Yang J., Hernandez B.J., Martinez Alanis D., del Narvaez Pilar O., Vila-Ellis L., Akiyama H., Evans S.E., Ostrin E.J., Chen J. (2016). The development and plasticity of alveolar type 1 cells. Development.

[31] Wan H., Xu Y., Ikegami M., Stahlman M.T., Kaestner K.H., Ang S.L., Whitsett J.A. (2004). Foxa2 is required for transition to air breathing at birth. Proc. Natl. Acad. Sci. USA.

[32] Ramirez M.I., Millien G., Hinds A., Cao Y., Seldin D.C., Williams M.C. (2003). T1alpha, a lung type I cell differentiation gene, is required for normal lung cell proliferation and alveolus formation at birth. Dev. Biol..

[33] Fernandez-Valdivia R., Zhang Y., Pai S., Metzker M.L., Schumacher A. (2006). l7Rn6 encodes a novel protein required for clara cell function in mouse lung development. Genetics.

[34] Hsu Y.C., Osinski J., Campbell C.E., Litwack E.D., Wang D., Liu S., Bachurski C.J., Gronostajski R.M. (2011). Mesenchymal nuclear factor I B regulates cell proliferation and epithelial differentiation during lung maturation. Dev. Biol..

[35] Wang I.C., Zhang Y., Snyder J., Sutherland M.J., Burhans M.S., Shannon J.M., Park H.J., Whitsett J.A., Kalinichenko V.V. (2010). Increased expression of FoxM1 transcription factor in respiratory epithelium inhibits lung sacculation and causes Clara cell hyperplasia. Dev. Biol..

[36] Yamamoto Y., Shiraishi I., Dai P., Hamaoka K., Takamatsu T. (2007). Regulation of embryonic lung vascular development by vascular endothelial growth factor receptors, Flk-1 and Flt-1. Anat. Rec..

[37] Bostrom H., Willetts K., Pekny M., Leveen P., Lindahl P., Hedstrand H., Pekna M., Hellstrom M., Gebre-Medhin S., Schalling M. (1996). PDGF-A signaling is a critical event in lung alveolar myofibroblast development and alveogenesis. Cell.

[38] Weinstein M., Xu X., Ohyama K., Deng C.X. (1998). FGFR-3 and FGFR-4 function cooperatively to direct alveogenesis in the murine lung. Development.

[39] Post L.C., Ternet M., Hogan B.L. (2000). Notch/Delta expression in the developing mouse lung. Mech. Dev..

[40] Morimoto M., Liu Z., Cheng H.T., Winters N., Bader D., Kopan R. (2010). Canonical Notch signaling in the developing lung is required for determination of arterial smooth muscle cells and selection of Clara versus ciliated cell fate. J. Cell Sci..

[41] Rao S.S., O’Neil J., Liberator C.D., Hardwick J.S., Dai X., Zhang T., Tyminski E., Yuan J., Kohl N.E., Richon V.M. (2009). Inhibition of NOTCH signaling by gamma secretase inhibitor engages the RB pathway and elicits cell cycle exit in T-cell acute lymphoblastic leukemia cells. Cancer Res..

[42] Kiyokawa H., Morimoto M. (2020). Notch signaling in the mammalian respiratory system, specifically the trachea and lungs, in development, homeostasis, regeneration, and disease. Dev. Growth Differ..

[43] Takeuchi H., Haltiwanger R.S. (2010). Role of glycosylation of Notch in development. Semin. Cell Dev. Biol..

[44] Tompkins D.H., Besnard V., Lange A.W., Wert S.E., Keiser A.R., Smith A.N., Lang R., Whitsett J.A. (2009). Sox2 is required for maintenance and differentiation of bronchiolar Clara, ciliated, and goblet cells. PLoS ONE.

[45] Zhang S., Loch A.J., Radtke F., Egan S.E., Xu K. (2013). Jagged1 is the major regulator of Notch-dependent cell fate in proximal airways. Dev. Dyn. Off. Publ. Am. Assoc. Anat..

[46] Dang T.P., Eichenberger S., Gonzalez A., Olson S., Carbone D.P. (2003). Constitutive activation of Notch3 inhibits terminal epithelial differentiation in lungs of transgenic mice. Oncogene.

[47] Tsao P.N., Wei S.C., Wu M.F., Huang M.T., Lin H.Y., Lee M.C., Lin K.M., Wang I.J., Kaartinen V., Yang L.T. (2011). Notch signaling prevents mucous metaplasia in mouse conducting airways during postnatal development. Development.

[48] Danahay H., Pessotti A.D., Coote J., Montgomery B.E., Xia D., Wilson A., Yang H., Wang Z., Bevan L., Thomas C. (2015). Notch2 is required for inflammatory cytokine-driven goblet cell metaplasia in the lung. Cell Rep..

[49] Lafkas D., Shelton A., Chiu C., de Leon Boenig G., Chen Y., Stawicki S.S., Siltanen C., Reichelt M., Zhou M., Wu X. (2015). Therapeutic antibodies reveal Notch control of transdifferentiation in the adult lung. Nature.

[50] Morimoto M., Nishinakamura R., Saga Y., Kopan R. (2012). Different assemblies of Notch receptors coordinate the distribution of the major bronchial Clara, ciliated and neuroendocrine cells. Development.

[51] Boucherat O., Chakir J., Jeannotte L. (2012). The loss of Hoxa5 function promotes Notch-dependent goblet cell metaplasia in lung airways. Biol. Open.

[52] Kuo C.S., Krasnow M.A. (2015). Formation of a Neurosensory Organ by Epithelial Cell Slithering. Cell.

[53] Noguchi M., Sumiyama K., Morimoto M. (2015). Directed Migration of Pulmonary Neuroendocrine Cells toward Airway Branches Organizes the Stereotypic Location of Neuroepithelial Bodies. Cell Rep..

[54] Stupnikov M.R., Yang Y., Mori M., Lu J., Cardoso W.V. (2019). Jagged and Delta-like ligands control distinct events during airway progenitor cell differentiation. ELife.

[55] Stanley P. (2007). Regulation of Notch signaling by glycosylation. Curr. Opin. Struct. Biol..

[56] Visan I., Yuan J.S., Liu Y., Stanley P., Guidos C.J. (2010). Lunatic fringe enhances competition for delta-like Notch ligands but does not overcome defective pre-TCR signaling during thymocyte beta-selection in vivo. J. Immunol..

[57] Yang L.T., Nichols J.T., Yao C., Manilay J.O., Robey E.A., Weinmaster G. (2005). Fringe glycosyltransferases differentially modulate Notch1 proteolysis induced by Delta1 and Jagged1. Mol. Biol. Cell.

[58] Liu Z., Brunskill E., Varnum-Finney B., Zhang C., Zhang A., Jay P.Y., Bernstein I., Morimoto M., Kopan R. (2015). The intracellular domains of Notch1 and Notch2 are functionally equivalent during development and carcinogenesis. Development.

[59] Lu T., Yang X., Huang Y., Zhao M., Li M., Ma K., Yin J., Zhan C., Wang Q. (2019). Trends in the incidence, treatment, and survival of patients with lung cancer in the last four decades. Cancer Manag. Res..

[60] Molina J.R., Yang P., Cassivi S.D., Schild S.E., Adjei A.A. (2008). Non-small cell lung cancer: Epidemiology, risk factors, treatment, and survivorship. Mayo Clin. Proc..

[61] Chen Z., Fillmore C.M., Hammerman P.S., Kim C.F., Wong K.K. (2014). Non-small-cell lung cancers: A heterogeneous set of diseases. Nat. Rev. Cancer.

[62] Ferone G., Song J.Y., Sutherland K.D., Bhaskaran R., Monkhorst K., Lambooij J.P., Proost N., Gargiulo G., Berns A. (2016). SOX2 Is the Determining Oncogenic Switch in Promoting Lung Squamous Cell Carcinoma from Different Cells of Origin. Cancer Cell.

[63] Xu X., Huang L., Futtner C., Schwab B., Rampersad R.R., Lu Y., Sporn T.A., Hogan B.L., Onaitis M.W. (2014). The cell of origin and subtype of K-Ras-induced lung tumors are modified by Notch and Sox2. Genes Dev..

[64] Spella M., Lilis I., Pepe M.A., Chen Y., Armaka M., Lamort A.S., Zazara D.E., Roumelioti F., Vreka M., Kanellakis N.I. (2019). Club cells form lung adenocarcinomas and maintain the alveoli of adult mice. ELife.

[65] Mainardi S., Mijimolle N., Francoz S., Vicente-Duenas C., Sanchez-Garcia I., Barbacid M. (2014). Identification of cancer initiating cells in K-Ras driven lung adenocarcinoma. Proc. Natl. Acad. Sci. USA.

[66] Sutherland K.D., Song J.Y., Kwon M.C., Proost N., Zevenhoven J., Berns A. (2014). Multiple cells-of-origin of mutant K-Ras-induced mouse lung adenocarcinoma. Proc. Natl. Acad. Sci. USA.

[67] Pagano P.C., Tran L.M., Bendris N., O’Byrne S., Tse H.T., Sharma S., Hoech J.W., Park S.J., Liclican E.L., Jing Z. (2017). Identification of a Human Airway Epithelial Cell Subpopulation with Altered Biophysical, Molecular, and Metastatic Properties. Cancer Prev. Res..

[68] Hanna J.M., Onaitis M.W. (2013). Cell of origin of lung cancer. J. Carcinog..

[69] Chen H.J., Poran A., Unni A.M., Huang S.X., Elemento O., Snoeck H.W., Varmus H. (2019). Generation of pulmonary neuroendocrine cells and SCLC-like tumors from human embryonic stem cells. J. Exp. Med..

[70] Sutherland K.D., Proost N., Brouns I., Adriaensen D., Song J.Y., Berns A. (2011). Cell of origin of small cell lung cancer: Inactivation of Trp53 and Rb1 in distinct cell types of adult mouse lung. Cancer Cell.

[71] Song H., Yao E., Lin C., Gacayan R., Chen M.H., Chuang P.T. (2012). Functional characterization of pulmonary neuroendocrine cells in lung development, injury, and tumorigenesis. Proc. Natl. Acad. Sci. USA.

[72] Park K.S., Liang M.C., Raiser D.M., Zamponi R., Roach R.R., Curtis S.J., Walton Z., Schaffer B.E., Roake C.M., Zmoos A.F. (2011). Characterization of the cell of origin for small cell lung cancer. Cell Cycle.

[73] Konishi J., Kawaguchi K.S., Vo H., Haruki N., Gonzalez A., Carbone D.P., Dang T.P. (2007). Gamma-secretase inhibitor prevents Notch3 activation and reduces proliferation in human lung cancers. Cancer Res..

[74] Zheng Y., de la Cruz C.C., Sayles L.C., Alleyne-Chin C., Vaka D., Knaak T.D., Bigos M., Xu Y., Hoang C.D., Shrager J.B. (2013). A rare population of CD24(+)ITGB4(+)Notch(hi) cells drives tumor propagation in NSCLC and requires Notch3 for self-renewal. Cancer Cell.

[75] Hassan K.A., Wang L., Korkaya H., Chen G., Maillard I., Beer D.G., Kalemkerian G.P., Wicha M.S. (2013). Notch pathway activity identifies cells with cancer stem cell-like properties and correlates with worse survival in lung adenocarcinoma. Clin. Cancer Res. Off. J. Am. Assoc. Cancer Res..

[76] Boareto M., Jolly M.K., Goldman A., Pietila M., Mani S.A., Sengupta S., Ben-Jacob E., Levine H., Onuchic J.N. (2016). Notch-Jagged signalling can give rise to clusters of cells exhibiting a hybrid epithelial/mesenchymal phenotype. J. R. Soc. Interface.

[77] Bocci F., Jolly M.K., Tripathi S.C., Aguilar M., Hanash S.M., Levine H., Onuchic J.N. (2017). Numb prevents a complete epithelial-mesenchymal transition by modulating Notch signalling. J. R. Soc. Interface.

[78] Westhoff B., Colaluca I.N., D’Ario G., Donzelli M., Tosoni D., Volorio S., Pelosi G., Spaggiari L., Mazzarol G., Viale G. (2009). Alterations of the Notch pathway in lung cancer. Proc. Natl. Acad. Sci. USA.

[79] Mimae T., Okada M., Hagiyama M., Miyata Y., Tsutani Y., Inoue T., Murakami Y., Ito A. (2012). Upregulation of notch2 and six1 is associated with progression of early-stage lung adenocarcinoma and a more aggressive phenotype at advanced stages. Clin. Cancer Res. Off. J. Am. Assoc. Cancer Res..

[80] Wang X., Meng Q., Qiao W., Ma R., Ju W., Hu J., Lu H., Cui J., Jin Z., Zhao Y. (2018). miR-181b/Notch2 overcome chemoresistance by regulating cancer stem cell-like properties in NSCLC. Stem Cell Res. Ther..

[81] Liu Z.Y., Wu T., Li Q., Wang M.C., Jing L., Ruan Z.P., Yao Y., Nan K.J., Guo H. (2016). Notch Signaling Components: Diverging Prognostic Indicators in Lung Adenocarcinoma. Medicine.

[82] Baumgart A., Mazur P.K., Anton M., Rudelius M., Schwamborn K., Feuchtinger A., Behnke K., Walch A., Braren R., Peschel C. (2015). Opposing role of Notch1 and Notch2 in a Kras(G12D)-driven murine non-small cell lung cancer model. Oncogene.

[83] Finn J., Sottoriva K., Pajcini K.V., Kitajewski J.K., Chen C., Zhang W., Malik A.B., Liu Y. (2019). Dlk1-Mediated Temporal Regulation of Notch Signaling Is Required for Differentiation of Alveolar Type II to Type I Cells during Repair. Cell Rep..

[84] Xu X., Rock J.R., Lu Y., Futtner C., Schwab B., Guinney J., Hogan B.L., Onaitis M.W. (2012). Evidence for type II cells as cells of origin of K-Ras-induced distal lung adenocarcinoma. Proc. Natl. Acad. Sci. USA.

[85] Ellisen L.W., Bird J., West D.C., Soreng A.L., Reynolds T.C., Smith S.D., Sklar J. (1991). TAN-1, the human homolog of the Drosophila notch gene, is broken by chromosomal translocations in T lymphoblastic neoplasms. Cell.

[86] Allen T.D., Zhu C.Q., Jones K.D., Yanagawa N., Tsao M.S., Bishop J.M. (2011). Interaction between MYC and MCL1 in the genesis and outcome of non-small-cell lung cancer. Cancer Res..

[87] Maraver A., Fernandez-Marcos P.J., Herranz D., Munoz-Martin M., Gomez-Lopez G., Canamero M., Mulero F., Megias D., Sanchez-Carbayo M., Shen J. (2012). Therapeutic effect of gamma-secretase inhibition in KrasG12V-driven non-small cell lung carcinoma by derepression of DUSP1 and inhibition of ERK. Cancer Cell.

[88] Licciulli S., Avila J.L., Hanlon L., Troutman S., Cesaroni M., Kota S., Keith B., Simon M.C., Pure E., Radtke F. (2013). Notch1 is required for Kras-induced lung adenocarcinoma and controls tumor cell survival via p53. Cancer Res..

[89] Eliasz S., Liang S., Chen Y., De Marco M.A., Machek O., Skucha S., Miele L., Bocchetta M. (2010). Notch-1 stimulates survival of lung adenocarcinoma cells during hypoxia by activating the IGF-1R pathway. Oncogene.

[90] Zou J., Li P., Lu F., Liu N., Dai J., Ye J., Qu X., Sun X., Ma D., Park J. (2013). Notch1 is required for hypoxia-induced proliferation, invasion and chemoresistance of T-cell acute lymphoblastic leukemia cells. J. Hematol. Oncol..

[91] Gately K., Forde L., Cuffe S., Cummins R., Kay E.W., Feuerhake F., O’Byrne K.J. (2014). High coexpression of both EGFR and IGF1R correlates with poor patient prognosis in resected non-small-cell lung cancer. Clin. Lung Cancer.

[92] Vilmar A., Santoni-Rugiu E., Cillas J.G., Huarriz M., Sorensen J.B. (2014). Insulin-like growth factor receptor 1 mRNA expression as a prognostic marker in advanced non-small cell lung cancer. Anticancer Res..

[93] Cai H., Lu W., Zhang Y., Liu H., Wang Z., Shen Y. (2019). Specific inhibition of Notch1 signaling suppresses properties of lung cancer stem cells. J. Cancer Res. Ther..

[94] Liu K.H., Tsai Y.T., Chin S.Y., Lee W.R., Chen Y.C., Shen S.C. (2018). Hypoxia Stimulates the Epithelial-to-Mesenchymal Transition in Lung Cancer Cells Through Accumulation of Nuclear beta-Catenin. Anticancer Res..

[95] Murakami A., Takahashi F., Nurwidya F., Kobayashi I., Minakata K., Hashimoto M., Nara T., Kato M., Tajima K., Shimada N. (2014). Hypoxia increases gefitinib-resistant lung cancer stem cells through the activation of insulin-like growth factor 1 receptor. PLoS ONE.

[96] Shao C., Sullivan J.P., Girard L., Augustyn A., Yenerall P., Rodriguez-Canales J., Liu H., Behrens C., Shay J.W., Wistuba I.I. (2014). Essential role of aldehyde dehydrogenase 1A3 for the maintenance of non-small cell lung cancer stem cells is associated with the STAT3 pathway. Clin. Cancer Res. Off. J. Am. Assoc. Cancer Res..

[97] Sullivan J.P., Spinola M., Dodge M., Raso M.G., Behrens C., Gao B., Schuster K., Shao C., Larsen J.E., Sullivan L.A. (2010). Aldehyde dehydrogenase activity selects for lung adenocarcinoma stem cells dependent on notch signaling. Cancer Res..

[98] Cerami E., Gao J., Dogrusoz U., Gross B.E., Sumer S.O., Aksoy B.A., Jacobsen A., Byrne C.J., Heuer M.L., Larsson E. (2012). The cBio cancer genomics portal: An open platform for exploring multidimensional cancer genomics data. Cancer Discov..

[99] Gao J., Aksoy B.A., Dogrusoz U., Dresdner G., Gross B., Sumer S.O., Sun Y., Jacobsen A., Sinha R., Larsson E. (2013). Integrative analysis of complex cancer genomics and clinical profiles using the cBioPortal. Sci. Signal..

[100] Chammaa M., Malysa A., Redondo C., Jang H., Chen W., Bepler G., Fernandez-Valdivia R. (2018). RUMI is a novel negative prognostic marker and therapeutic target in non-small-cell lung cancer. J. Cell. Physiol..

[101] Arasada R.R., Amann J.M., Rahman M.A., Huppert S.S., Carbone D.P. (2014). EGFR blockade enriches for lung cancer stem-like cells through Notch3-dependent signaling. Cancer Res..

[102] Arasada R.R., Shilo K., Yamada T., Zhang J., Yano S., Ghanem R., Wang W., Takeuchi S., Fukuda K., Katakami N. (2018). Notch3-dependent beta-catenin signaling mediates EGFR TKI drug persistence in EGFR mutant NSCLC. Nature Commun..

[103] Ali S.A., Justilien V., Jamieson L., Murray N.R., Fields A.P. (2016). Protein Kinase Ciota Drives a NOTCH3-dependent Stem-like Phenotype in Mutant KRAS Lung Adenocarcinoma. Cancer Cell.

[104] Yagci E., Degirmenci I., Ozbayer C., Ak G., Saydam F., Metintas M. (2019). Common Variants rs3815188 and rs1043994 on Notch3 Gene Confer Susceptibility to Lung Cancer: A Hospital-Based Case-Control Study. J. Environ. Pathol. Toxicol. Oncol. Off. Organ Int. Soc. Environ. Toxicol. Cancer.

[105] Xu Q.P., Xiao R.D., Xiong W.M., He F., Cai L. (2018). Association between polymorphism in notch signaling pathway and lung cancer risk. Zhonghua Yu Fang Yi Xue Za Zhi [Chin. J. Prev. Med. ].

[106] George J., Lim J.S., Jang S.J., Cun Y., Ozretic L., Kong G., Leenders F., Lu X., Fernandez-Cuesta L., Bosco G. (2015). Comprehensive genomic profiles of small cell lung cancer. Nature.

[107] Augert A., Eastwood E., Ibrahim A.H., Wu N., Grunblatt E., Basom R., Liggitt D., Eaton K.D., Martins R., Poirier J.T. (2019). Targeting NOTCH activation in small cell lung cancer through LSD1 inhibition. Sci. Signal..

[108] Gordian E., Gimbrone N., Pannuti A., Miele L., Cress W.D., Muñoz-Antonia T. (2017). Abstract 4456: Novel oncogenic function of Notch4 in Hispanic lung cancer. Cancer Res..

[109] Xu S., Zhao Q., Wei S., Wu Y., Liu J., Shi T., Zhou Q., Chen J. (2015). Next Generation Sequencing Uncovers Potential Genetic Driver Mutations of Malignant Pulmonary Granular Cell Tumor. J. Thorac. Oncol. Off. Publ. Int. Assoc. Study Lung Cancer.

[110] Mutvei A.P., Fredlund E., Lendahl U. (2015). Frequency and distribution of Notch mutations in tumor cell lines. BMC Cancer.

[111] Pancewicz-Wojtkiewicz J., Eljaszewicz A., Kowalczuk O., Niklinska W., Charkiewicz R., Kozlowski M., Miasko A., Moniuszko M. (2017). Prognostic significance of Notch ligands in patients with non-small cell lung cancer. Oncol. Lett..

[112] Gao Y., Ge G., Ji H. (2011). LKB1 in lung cancerigenesis: A serine/threonine kinase as tumor suppressor. Protein Cell.

[113] Shah U., Sharpless N.E., Hayes D.N. (2008). LKB1 and lung cancer: More than the usual suspects. Cancer Res..

[114] Han X., Li F., Fang Z., Gao Y., Li F., Fang R., Yao S., Sun Y., Li L., Zhang W. (2014). Transdifferentiation of lung adenocarcinoma in mice with Lkb1 deficiency to squamous cell carcinoma. Nat. Commun..

[115] Ji H., Ramsey M.R., Hayes D.N., Fan C., McNamara K., Kozlowski P., Torrice C., Wu M.C., Shimamura T., Perera S.A. (2007). LKB1 modulates lung cancer differentiation and metastasis. Nature.

[116] Jackson E.L., Willis N., Mercer K., Bronson R.T., Crowley D., Montoya R., Jacks T., Tuveson D.A. (2001). Analysis of lung tumor initiation and progression using conditional expression of oncogenic K-ras. Genes Dev..

[117] Skoulidis F., Goldberg M.E., Greenawalt D.M., Hellmann M.D., Awad M.M., Gainor J.F., Schrock A.B., Hartmaier R.J., Trabucco S.E., Gay L. (2018). STK11/LKB1 Mutations and PD-1 Inhibitor Resistance in KRAS-Mutant Lung Adenocarcinoma. Cancer Discov..

[118] Rodon L., Svensson R.U., Wiater E., Chun M.G.H., Tsai W.W., Eichner L.J., Shaw R.J., Montminy M. (2019). The CREB coactivator CRTC2 promotes oncogenesis in LKB1-mutant non-small cell lung cancer. Sci. Adv..

[119] Sikder H.A., Devlin M.K., Dunlap S., Ryu B., Alani R.M. (2003). Id proteins in cell growth and tumorigenesis. Cancer Cell.

[120] Chen D., Forootan S.S., Gosney J.R., Forootan F.S., Ke Y. (2014). Increased expression of Id1 and Id3 promotes tumorigenicity by enhancing angiogenesis and suppressing apoptosis in small cell lung cancer. Genes Cancer.

[121] Bai G., Sheng N., Xie Z., Bian W., Yokota Y., Benezra R., Kageyama R., Guillemot F., Jing N. (2007). Id sustains Hes1 expression to inhibit precocious neurogenesis by releasing negative autoregulation of Hes1. Dev. Cell.

[122] Wang H.C., Perry S.S., Sun X.H. (2009). Id1 attenuates Notch signaling and impairs T-cell commitment by elevating Deltex1 expression. Mol. Cell. Biol..

[123] Hu B.D., Guo J., Ye Y.Z., Du T., Cheng C.S., Jiang Q., Liu R.N., Zhang Y.B. (2018). Specific inhibitor of Notch3 enhances the sensitivity of NSCLC cells to gemcitabine. Oncol. Rep..

[124] Inge L.J., Coon K.D., Smith M.A., Bremner R.M. (2009). Expression of LKB1 tumor suppressor in non-small cell lung cancer determines sensitivity to 2-deoxyglucose. J. Thorac. Cardiovasc. Surg..

[125] Roman M., Baraibar I., Lopez I., Nadal E., Rolfo C., Vicent S., Gil-Bazo I. (2018). KRAS oncogene in non-small cell lung cancer: Clinical perspectives on the treatment of an old target. Mol. Cancer.

[126] Black R.C., Khurshid H. (2015). NSCLC: An Update of Driver Mutations, Their Role in Pathogenesis and Clinical Significance. Rhode Isl. Med J..

[127] Sosa Iglesias V., Giuranno L., Dubois L.J., Theys J., Vooijs M. (2018). Drug Resistance in Non-Small Cell Lung Cancer: A Potential for NOTCH Targeting?. Front. Oncol..

[128] Espinoza I., Miele L. (2013). Notch inhibitors for cancer treatment. Pharmacol. Ther..

[129] Katoh M., Katoh M. (2020). Precision medicine for human cancers with Notch signaling dysregulation. Int. J. Mol. Med..

[130] Takebe N., Nguyen D., Yang S.X. (2014). Targeting notch signaling pathway in cancer: Clinical development advances and challenges. Pharmacol. Ther..

[131] Liu J., Mao Z., Huang J., Xie S., Liu T., Mao Z. (2014). Blocking the NOTCH pathway can inhibit the growth of CD133-positive A549 cells and sensitize to chemotherapy. Biochem. Biophys. Res. Commun..

[132] Sosa Iglesias V., Theys J., Groot A.J., Barbeau L.M.O., Lemmens A., Yaromina A., Losen M., Houben R., Dubois L., Vooijs M. (2018). Synergistic Effects of NOTCH/gamma-Secretase Inhibition and Standard of Care Treatment Modalities in Non-small Cell Lung Cancer Cells. Front. Oncol..

[133] Carroll C.M., Li Y.M. (2016). Physiological and pathological roles of the gamma-secretase complex. Brain Res. Bull..

[134] Xie M., He C.S., Wei S.H., Zhang L. (2013). Notch-1 contributes to epidermal growth factor receptor tyrosine kinase inhibitor acquired resistance in non-small cell lung cancer in vitro and in vivo. Eur. J. Cancer.

[135] Morgan K.M., Fischer B.S., Lee F.Y., Shah J.J., Bertino J.R., Rosenfeld J., Singh A., Khiabanian H., Pine S.R. (2017). Gamma Secretase Inhibition by BMS-906024 Enhances Efficacy of Paclitaxel in Lung Adenocarcinoma. Mol. Cancer Ther..

[136] Xie M., He J., He C., Wei S. (2015). gamma Secretase inhibitor BMS-708163 reverses resistance to EGFR inhibitor via the PI3K/Akt pathway in lung cancer. J. Cell. Biochem..

[137] Ambrogio C., Gomez-Lopez G., Falcone M., Vidal A., Nadal E., Crosetto N., Blasco R.B., Fernandez-Marcos P.J., Sanchez-Cespedes M., Ren X. (2016). Combined inhibition of DDR1 and Notch signaling is a therapeutic strategy for KRAS-driven lung adenocarcinoma. Nat. Med..

[138] O’Neil N.J., Bailey M.L., Hieter P. (2017). Synthetic lethality and cancer. Nat. Rev. Genet..

[139] Luo S.Y., Lam D.C. (2013). Oncogenic driver mutations in lung cancer. Transl. Respir. Med..

[140] Liu Y., Sun L., Xiong Z.C., Sun X., Zhang S.L., Ma J.T., Han C.B. (2017). Meta-analysis of the impact of de novo and acquired EGFR T790M mutations on the prognosis of patients with non-small cell lung cancer receiving EGFR-TKIs. OncoTargets Ther..

[141] Schnute B., Troost T., Klein T. (2018). Endocytic Trafficking of the Notch Receptor. Adv. Exp. Med. Biol..

[142] Steinbuck M.P., Winandy S. (2018). A Review of Notch Processing With New Insights Into Ligand-Independent Notch Signaling in T-Cells. Front. Immunol..

[143] Conner S.D. (2016). Regulation of Notch Signaling Through Intracellular Transport. Int. Rev. Cell Mol. Biol..

[144] Palmer W.H., Deng W.M. (2015). Ligand-Independent Mechanisms of Notch Activity. Trends Cell Biol..

[145] Fortini M.E., Bilder D. (2009). Endocytic regulation of Notch signaling. Curr. Opin. Genet. Dev..

[146] Portela M., Yang L., Paul S., Li X., Veraksa A., Parsons L.M., Richardson H.E. (2018). Lgl reduces endosomal vesicle acidification and Notch signaling by promoting the interaction between Vap33 and the V-ATPase complex. Sci. Signal..

[147] Gallagher C.M., Knoblich J.A. (2006). The conserved c2 domain protein lethal (2) giant discs regulates protein trafficking in Drosophila. Dev. Cell.

[148] Jaekel R., Klein T. (2006). The Drosophila Notch inhibitor and tumor suppressor gene lethal (2) giant discs encodes a conserved regulator of endosomal trafficking. Dev. Cell.

[149] Childress J.L., Acar M., Tao C., Halder G. (2006). Lethal giant discs, a novel C2-domain protein, restricts notch activation during endocytosis. Curr. Biol. CB.

[150] Brumby A.M., Richardson H.E. (2003). scribble mutants cooperate with oncogenic Ras or Notch to cause neoplastic overgrowth in Drosophila. EMBO J..

[151] Paul M.S., Singh A., Dutta D., Mutsuddi M., Mukherjee A. (2018). Notch signals modulate lgl mediated tumorigenesis by the activation of JNK signaling. BMC Res. Notes.

[152] Li Q., Shen L., Xin T., Xiang W., Chen W., Gao Y., Zhu M., Yu L., Li M. (2009). Role of Scrib and Dlg in anterior-posterior patterning of the follicular epithelium during Drosophila oogenesis. BMC Dev. Biol..

[153] Kobia F., Duchi S., Deflorian G., Vaccari T. (2014). Pharmacologic inhibition of vacuolar H+ ATPase reduces physiologic and oncogenic Notch signaling. Mol. Oncol..

[154] Pinazza M., Ghisi M., Minuzzo S., Agnusdei V., Fossati G., Ciminale V., Pezze L., Ciribilli Y., Pilotto G., Venturoli C. (2018). Histone deacetylase 6 controls Notch3 trafficking and degradation in T-cell acute lymphoblastic leukemia cells. Oncogene.

[155] Acar M., Jafar-Nejad H., Takeuchi H., Rajan A., Ibrani D., Rana N.A., Pan H., Haltiwanger R.S., Bellen H.J. (2008). Rumi is a CAP10 domain glycosyltransferase that modifies Notch and is required for Notch signaling. Cell.

[156] Leonardi J., Fernandez-Valdivia R., Li Y.D., Simcox A.A., Jafar-Nejad H. (2011). Multiple O-glucosylation sites on Notch function as a buffer against temperature-dependent loss of signaling. Development.

[157] Bender M.H., Gao H., Capen A.R., Clay J.M., Hipskind P.A., Reel J.K., Zamek-Gliszczynski M.J., Manro J.R., Benhadji K., Patel B.K.R. (2013). Abstract 1131: Novel inhibitor of Notch signaling for the treatment of cancer. Cancer Res..

[158] Massard C., Azaro A., Soria J.C., Lassen U., Le Tourneau C., Sarker D., Smith C., Ohnmacht U., Oakley G., Patel B.K.R. (2018). First-in-human study of LY3039478, an oral Notch signaling inhibitor in advanced or metastatic cancer. Ann. Oncol. Off. J. Eur. Soc. Med Oncol..

[159] Mir O., Azaro A., Merchan J., Chugh R., Trent J., Rodon J., Ohnmacht U., Diener J.T., Smith C., Yuen E. (2018). Notch pathway inhibition with LY3039478 in soft tissue sarcoma and gastrointestinal stromal tumours. Eur. J. Cancer.

[160] Luistro L., He W., Smith M., Packman K., Vilenchik M., Carvajal D., Roberts J., Cai J., Berkofsky-Fessler W., Hilton H. (2009). Preclinical profile of a potent gamma-secretase inhibitor targeting notch signaling with in vivo efficacy and pharmacodynamic properties. Cancer Res..

[161] Diaz-Padilla I., Hirte H., Oza A.M., Clarke B.A., Cohen B., Reedjik M., Zhang T., Kamel-Reid S., Ivy S.P., Hotte S.J. (2013). A phase Ib combination study of RO4929097, a gamma-secretase inhibitor, and temsirolimus in patients with advanced solid tumors. Investig. New Drugs.

[162] Sahebjam S., Bedard P.L., Castonguay V., Chen Z., Reedijk M., Liu G., Cohen B., Zhang W.J., Clarke B., Zhang T. (2013). A phase I study of the combination of ro4929097 and cediranib in patients with advanced solid tumours (PJC-004/NCI 8503). Br. J. Cancer.

[163] Pant S., Jones S.F., Kurkjian C.D., Infante J.R., Moore K.N., Burris H.A., McMeekin D.S., Benhadji K.A., Patel B.K.R., Frenzel M.J. (2016). A first-in-human phase I study of the oral Notch inhibitor, LY900009, in patients with advanced cancer. Eur. J. Cancer.

[164] Messersmith W.A., Shapiro G.I., Cleary J.M., Jimeno A., Dasari A., Huang B., Shaik M.N., Cesari R., Zheng X., Reynolds J.M. (2015). A Phase I, dose-finding study in patients with advanced solid malignancies of the oral gamma-secretase inhibitor PF-03084014. Clin. Cancer Res. Off. J. Am. Assoc. Cancer Res..

[165] Kummar S., O’Sullivan Coyne G., Do K.T., Turkbey B., Meltzer P.S., Polley E., Choyke P.L., Meehan R., Vilimas R., Horneffer Y. (2017). Clinical Activity of the gamma-Secretase Inhibitor PF-03084014 in Adults With Desmoid Tumors (Aggressive Fibromatosis). J. Clin. Oncol. Off. J. Am. Soc. Clin. Oncol..

[166] McKeage M.J., Kotasek D., Markman B., Hidalgo M., Millward M.J., Jameson M.B., Harris D.L., Stagg R.J., Kapoun A.M., Xu L. (2018). Phase IB Trial of the Anti-Cancer Stem Cell DLL4-Binding Agent Demcizumab with Pemetrexed and Carboplatin as First-Line Treatment of Metastatic Non-Squamous NSCLC. Target. Oncol..

[167] Rudin C.M., Pietanza M.C., Bauer T.M., Ready N., Morgensztern D., Glisson B.S., Byers L.A., Johnson M.L., Burris H.A., Robert F. (2017). Rovalpituzumab tesirine, a DLL3-targeted antibody-drug conjugate, in recurrent small-cell lung cancer: A first-in-human, first-in-class, open-label, phase 1 study. Lancet. Oncol..

[168] Morgensztern D., Besse B., Greillier L., Santana-Davila R., Ready N., Hann C.L., Glisson B.S., Farago A.F., Dowlati A., Rudin C.M. (2019). Efficacy and Safety of Rovalpituzumab Tesirine in Third-Line and Beyond Patients with DLL3-Expressing, Relapsed/Refractory Small-Cell Lung Cancer: Results From the Phase II TRINITY Study. Clin. Cancer Res. Off. J. Am. Assoc. Cancer Res..

[169] OncoMed Pharmaceuticals, Inc. (2017). A Phase 1b/2 Study of OMP-59R5 (Tarextumab) in Combination with Etoposide and Platinum Therapy.

[170] Chiorean E.G., LoRusso P., Strother R.M., Diamond J.R., Younger A., Messersmith W.A., Adriaens L., Liu L., Kao R.J., DiCioccio A.T. (2015). A Phase I First-in-Human Study of Enoticumab (REGN421), a Fully Human Delta-like Ligand 4 (Dll4) Monoclonal Antibody in Patients with Advanced Solid Tumors. Clin. Cancer Res. Off. J. Am. Assoc. Cancer Res..

[171] Lim J.S., Ibaseta A., Fischer M.M., Cancilla B., O’Young G., Cristea S., Luca V.C., Yang D., Jahchan N.S., Hamard C. (2017). Intratumoural heterogeneity generated by Notch signalling promotes small-cell lung cancer. Nature.

[172] Sriuranpong V., Borges M.W., Ravi R.K., Arnold D.R., Nelkin B.D., Baylin S.B., Ball D.W. (2001). Notch signaling induces cell cycle arrest in small cell lung cancer cells. Cancer Res..

[173] Soda M., Choi Y.L., Enomoto M., Takada S., Yamashita Y., Ishikawa S., Fujiwara S., Watanabe H., Kurashina K., Hatanaka H. (2007). Identification of the transforming EML4-ALK fusion gene in non-small-cell lung cancer. Nature.

[174] Li Y., Ye X., Liu J., Zha J., Pei L. (2011). Evaluation of EML4-ALK fusion proteins in non-small cell lung cancer using small molecule inhibitors. Neoplasia.

[175] Kogita A., Togashi Y., Hayashi H., Sogabe S., Terashima M., De Velasco M.A., Sakai K., Fujita Y., Tomida S., Takeyama Y. (2014). Hypoxia induces resistance to ALK inhibitors in the H3122 non-small cell lung cancer cell line with an ALK rearrangement via epithelial-mesenchymal transition. Int. J. Oncol..

[176] Larose H., Prokoph N., Matthews J.D., Schlederer M., Hogler S., Alsulami A.F., Ducray S.P., Nuglozeh E., Fazaludeen F.M.S., Elmouna A. (2020). Whole Exome Sequencing reveals NOTCH1 mutations in anaplastic large cell lymphoma and points to Notch both as a key pathway and a potential therapeutic target. Haematologica.

[177] Canon J., Rex K., Saiki A.Y., Mohr C., Cooke K., Bagal D., Gaida K., Holt T., Knutson C.G., Koppada N. (2019). The clinical KRAS(G12C) inhibitor AMG 510 drives anti-tumour immunity. Nature.

[178] Hallin J., Engstrom L.D., Hargis L., Calinisan A., Aranda R., Briere D.M., Sudhakar N., Bowcut V., Baer B.R., Ballard J.A. (2020). The KRAS(G12C) Inhibitor MRTX849 Provides Insight toward Therapeutic Susceptibility of KRAS-Mutant Cancers in Mouse Models and Patients. Cancer Discov..

[179] Munoz-Maldonado C., Zimmer Y., Medova M. (2019). A Comparative Analysis of Individual RAS Mutations in Cancer Biology. Front. Oncol..

[180] Park S., Kim J.Y., Lee S.H., Suh B., Keam B., Kim T.M., Kim D.W., Heo D.S. (2017). KRAS G12C mutation as a poor prognostic marker of pemetrexed treatment in non-small cell lung cancer. Korean J. Intern. Med..

[181] Dexter J.S. (1914). The Analysis of a Case of Continuous Variation in Drosophila by a Study of Its Linkage Relations. Am. Nat..

[182] Morgan T.H. (1917). The Theory of the Gene. Am. Nat..

[183] Morgan T.H. (1928). The Theory of the Gene.

[184] Poulson D.F. (1937). Chromosomal Deficiencies and the Embryonic Development of Drosophila Melanogaster. Proc. Natl. Acad. Sci. USA.

[185] Kidd S., Kelley M.R., Young M.W. (1986). Sequence of the notch locus of Drosophila melanogaster: Relationship of the encoded protein to mammalian clotting and growth factors. Mol. Cell. Biol..

[186] Wharton K.A., Johansen K.M., Xu T., Artavanis-Tsakonas S. (1985). Nucleotide sequence from the neurogenic locus notch implies a gene product that shares homology with proteins containing EGF-like repeats. Cell.

